# How to explore within-person and between-person measurement model differences in intensive longitudinal data with the R package *lmfa*

**DOI:** 10.3758/s13428-022-01898-1

**Published:** 2022-09-01

**Authors:** Leonie V. D. E. Vogelsmeier, Jeroen K. Vermunt, Kim De Roover

**Affiliations:** Department of Methodology and Statistics, Tilburg University, PO Box 90153, 5000 LE Tilburg, The Netherlands

**Keywords:** Intensive longitudinal data, ESM, Measurement invariance, Factor analysis, Latent Markov modeling, Three-step approach, R, Software package

## Abstract

Intensive longitudinal data (ILD) have become popular for studying within-person dynamics in psychological constructs (or between-person differences therein). Before investigating the dynamics, it is crucial to examine whether the measurement model (MM) is the same across subjects and time and, thus, whether the measured constructs have the same meaning. If the MM differs (e.g., because of changes in item interpretation or response styles), observations cannot be validly compared. Exploring differences in the MM for ILD can be done with latent Markov factor analysis (LMFA), which classifies observations based on the underlying MM (for many subjects and time points simultaneously) and thus shows which observations are comparable. However, the complexity of the method or the fact that no open-source software for LMFA existed until now may have hindered researchers from applying the method in practice. In this article, we provide a step-by-step tutorial for the new user-friendly software package *lmfa*, which allows researchers to easily perform the analysis LMFA in the freely available software R to investigate MM differences in their own ILD.

**Supplementary Information:**

The online version contains supplementary material available at 10.3758/s13428-022-01898-1.

## Introduction

In recent years, researchers have shown an increased interest in intensive longitudinal data (ILD) for studying the dynamics of one or more latent psychological constructs (or “factors”) such as depression or affective well-being for many subjects over a longer time (e.g., >30 measurement occasions; Asparouhov et al., 2017). The ILD are commonly obtained using experience sampling methodology (ESM; Scollon et al., 2003), where multiple subjects repeatedly complete small questionnaires—containing items intended to measure one or more latent factors—at random (or event-based) time points, several times a day for several days or weeks via a smartphone app. State-of-the-art analyses to model dynamics in psychological factors for many subjects over time range from basic random-effect models (for studying individual differences in the dynamics or average levels of the factors; Hamaker et al., 2015; Myin-Germeys et al., 2018), over multilevel autoregressive models (for studying individual differences in lagged relationships between factors; e.g., Bringmann et al., 2013), to the advanced dynamic structural equation modeling framework that allows for the estimation of more complex models (e.g., models containing multiple outcome variables; McNeish & Hamaker, 2020). Furthermore, various (mixture) variants of latent growth models (Muthén, 2002) and latent Markov models (Bartolucci et al., 2015; Baum et al., 1970; Vermunt et al., 1999; Wiggins, 1973) are used to study individual-level change and discrete changes at subsequent time points over time, respectively.

While the technology for gathering ILD and approaches for analyzing dynamics in the measured constructs are readily available, an important point of concern for many researchers before they start their analyses is whether the latent factors have the same meaning across subjects and time points and, thus, whether observations are comparable. For this, the measurement model (MM) needs to be invariant across observations; that is, measurement invariance (MI) must hold. The MM indicates which factors are measured by which indicators and, for continuous item responses, is traditionally obtained with factor analysis (FA; Lawley & Maxwell, 1962). In the resulting MM (or “FA model”), factor loadings indicate the extent to which items measure the factors, and item intercepts indicate the expected item scores when scores on the factors are equal to zero. If the loadings, the intercepts, or the number of factors differ within or across subjects, MI is violated, and factors cannot be meaningfully compared (Adolf et al., 2014). However, invariance within and between subjects is easily violated because of differences and changes in response styles (Moors, 2003; Paulhus, 1991) or item interpretations (Oort et al., 2005). Thus, the MM may differ across subjects and change over time.

To clarify the possible non-invariance of MMs, consider the following example. Researchers conduct an ESM study to investigate between-subject differences regarding dynamics in the affective well-being of adolescents in different contexts. On the one hand, the underlying MM may differ across adolescents because people generally differ in their ability to label emotions in a granular way (Barrett et al., 2001; Erbas et al., 2020; Kashdan et al., 2015). The “high differentiators” differentiate more between specific emotions such as feeling content or happy than the “low differentiators”, who focus more on the valence of a feeling and, thus, whether an emotion is positive or negative (Barrett, 1998; Erbas et al., 2015). A result could be that several factors underlie the responses of the high differentiators (say, four factors pertaining to high- and low-arousal positive and negative affect). In contrast, only one factor underlies the responses of the low differentiators (say, a bipolar “valence of affect” factor).

On the other hand, the MM may change within adolescents over time. For instance, adolescents who are generally high differentiators may also switch to a MM with a single “valence of affect” factor when being exposed to a stressful situation (e.g., right before an exam) because stress triggers a valence focus (Erbas et al., 2018). Because the low differentiators respond according to a single valence of affect factor, regardless of experienced stress, the same MM would be underlying their responses during the entire participation.

Undetected measurement non-invariance is a threat to valid inferences from ILD analyses. Therefore, detecting non-invariance is crucial. Until recently, researchers could only test whether the MM is invariant across (groups of) subjects and/or time points (e.g., using traditional MI tests that are available in the R package *lavaan*; Rosseel, 2012). One limitation is that it is only possible to investigate non-invariance across subjects (assuming invariance over time) or to investigate invariance over time (assuming invariance across subjects) and not to investigate both at the same time. Furthermore, if the results indicate that invariance is untenable across subjects and/or time points, researchers cannot identify for which subjects or time points the MMs differ and what the different MMs look like without conducting pairwise comparisons of the subject- or time-point-specific MM parameters. This quickly becomes infeasible for ILD, which usually contain many observations from many subjects.

These above-described problems were solved by Vogelsmeier et al. (2019b), who developed latent Markov factor analysis (LMFA). LMFA allows researchers to conveniently explore all kinds of MM differences across subjects and time in ILD, in which latent constructs are measured with items on a continuous scale^1^. LMFA is a mixture modeling approach that combines a latent Markov model (LMM; Bartolucci et al., 2014; Collins & Lanza, 2010) with mixture FA (McLachlan & Peel, 2000; McNicholas, 2016): First, the LMM clusters observations according to their underlying MM into a few dynamic latent states. The latent states are equivalent to latent classes in a latent class analysis or mixture model but are called states in an LMM because subjects can transition between latent classes over time. Second, FA reveals what the underlying MMs look like for each state.

In summary, LMFA classifies observations into different states pertaining to different MMs, and invariance holds for observations in the same state but is violated for observations in different states. Within-person invariance holds for subjects that are in the same state throughout their participation, and between-person invariance holds if the state is also the same across subjects. Researchers can then decide how to continue with their data analysis (e.g., retaining observations from one state or removing non-invariant items; see “Proceeding based on the results of LMFA” section for a more elaborate discussion). Researchers can also learn from subjects’ transitions between MMs by including time-varying or time-constant covariates as predictors of the state memberships (e.g., “stress” could be included when analyzing the changes in the MM in our adolescent example).

By applying LMFA to ILD, we can thus answer the following questions: (1) *How many MMs are underlying our ILD?* (2) *How do the MMs differ?* (3) *How do subjects transition between the MMs over time, and is this related to time- or subject-specific covariates?* (4) *For which subjects does within-person invariance hold over time, and for which of these subjects does between-person invariance hold?*

More and more researchers are eager to explore MI in their ILD (Horstmann & Ziegler, 2020). However, until now, LMFA was only available in the commercial software Latent GOLD (LG; Vermunt & Magidson, 2016), and, thus, not all researchers had access to the novel method. This has now changed with the release of the package *lmfa* (Vogelsmeier & De Roover, 2021), which allows researchers to perform all necessary steps in the open-source software R (R Core Team, 2020) and which is available on GitHub (https://github.com/LeonieVm/lmfa). This paper provides a tutorial for the *lmfa* package that guides users such as applied researchers through the steps of performing the analysis and interpreting the results to increase researchers’ confidence and ease in using LMFA. The tutorial targets an audience with a basic understanding of R but not necessarily of the LMFA model. The technical details of the *lmfa* package are presented in the online Supplementary Material. However, understanding the technical details is not relevant for understanding the method and following the tutorial.

The remainder of this paper is organized as follows: First, in “Illustrative example” section, we introduce an example dataset. Then, in “Recap latent Markov factor analysis” section, we recap the LMFA method and explain how it is estimated in *lmfa*. Then, in “How to conduct LMFA with the *lmfa* package” section, we guide the reader through the different analysis steps by means of annotated R code. Next, in “Proceeding based on the results of LMFA” section, we describe how to proceed with ILD analyses based on the results of LMFA. Finally, in “Discussion” section, we conclude with a discussion about current limitations and possible future extensions of *lmfa*.

## Illustrative example

To clarify the data structure, consider the following example dataset that will be used throughout this tutorial. The data is a synthetic dataset inspired by a real ESM dataset, which was used in Vogelsmeier et al. (2019b) to illustrate how to explore MM changes by means of LMFA without covariates. Every evening for about three months, multiple subjects (suffering from anhedonia, one of the core symptoms of depression; Van Roekel et al., 2017) reported their affect and the unpleasantness of the most unpleasant event they experienced since the previous measurement occasion (in the following, just “negative event”). Affect was measured with ten positive affect items (“interested”, “joyful”, “determined”, “calm”, “lively”, “enthusiastic”, “relaxed”, “cheerful”, “content”, and “energetic”) and eight negative affect items (“upset”, “gloomy”, “sluggish”, “anxious”, “bored”, “irritated”, “nervous”, and “listless”), and a single item was used to assess the negative event. All items were assessed on a visual analogue scale ranging from 0 = “Not at all” to 100 = “Very much”. Moreover, after the first month, subjects were randomly assigned to receive an intervention to reduce anhedonia or not.^2^ The results of LMFA indicated that most subjects transitioned between three MMs that differed with regard to the number and nature of the factors. Descriptive statistics showed a relation between the states and the two covariates “had an intervention” and “negative event”.

For the tutorial in this article, we created a dataset with MMs similar to the ones found in the real data application (but somewhat adjusted and simplified) and with the two time-varying covariates “had an intervention” (coded as 1 = “yes” and 0 = “no”) and “negative event” affecting the transitions between the states. The dataset contains data for 100 subjects with a mean of 47.76 observations and an SD of 6.56, resulting in a total number of observations equal to 4776. The intervals between measurement occasions differ within and across subjects, with an average length of 1.22 days and an SD of 1.02. The negative event scores vary within and across subjects, with a mean of 49.65 and an SD of 15.11. Of all subjects, 50 receive no intervention, and 50 receive one intervention after approximately one third of their total participation duration. Throughout the tutorial, the dataset will be used to show how the different analysis steps answer the research questions about MM differences and changes (see “Introduction” section for research questions 1–4). Note that the true number of states and factors and the relevant covariates (i.e., as in the data-generating model) are not known in empirical practice. The required model selection and covariate selection procedures are explained as part of the tutorial in “BIC and CHull” and “Covariate selection procedure using Wald tests” sections, respectively.

## Recap latent Markov factor analysis

The LMFA method was introduced by Vogelsmeier et al. (2019b) and was further extended by Vogelsmeier et al. (2019a, 2021a). In this section, we summarize the relevant information in non-technical terms. A corresponding summary of the technical details is provided in the Supplementary Material. LMFA consists of two building blocks. The first one pertains to the state-specific MMs and thus to an FA model for each state that specifies which constructs are measured by which items. The second building block is the LMM, which models the transitions between MMs over time (Bartolucci et al., 2015; Zucchini et al., 2016). Note that there are two types of LMMs. First, the discrete-time (DT)-LMM (Bartolucci et al., 2015; Zucchini et al., 2016) assumes equal intervals across subjects and time. In contrast, the continuous-time (CT)-LMM (Böckenholt, 2005; Jackson & Sharples, 2002) accommodates unequally spaced observations, which is usually more realistic in ILD (e.g., due to random beeps or skipped measurement occasions). However, the CT-LMM also works for equal intervals. In fact, estimating a CT-LMM with equal intervals is similar to estimating a DT-LMM, but the parameter interpretation differs (Vermunt & Magidson, 2016), which will be clarified in “The transition model” section. The *lmfa* package uses CT-LMM because it is more generally applicable.

LMFA can be estimated with a full information maximum likelihood (FIML) estimation (Vogelsmeier et al., 2019a, b) or with a three-step (3S) estimation (Vogelsmeier et al., 2021a). The latter breaks down the estimation of LMFA into three steps. Although the 3S approach works slightly less well in assigning observations to the correct state (and thus MM) than the FIML estimation, the 3S approach is preferred when investigating covariate effects because it ensures that misspecifications of covariate effects do not falsify the formation of the MMs. Therefore, *lmfa* uses the 3S estimation, which will be explained below (“Estimation” section).^3^

### The state-specific measurement models

In LMFA, the MMs are determined by state-specific FA models, which consist of three types of parameters. Depending on which parameters differ across states, different levels of MI are violated. The first type of parameter is the factor loadings, which determine the item–factor relations and, hence, the degree to which an item measures a factor or, stated differently, to what extent an item is predicted by the underlying factor. Thus, items with stronger loadings are better measures of a factor than items with lower loadings. Second, item intercepts are the expected scores for an item when the factor scores are equal to zero. Third, the items’ unique variances indicate the variance of an item that is unique to the item and, hence, that is not explained by the factors (for the mathematical notation and the technical details, see Supplementary Material S.2.2). The three types of parameters can take on different values across states and inform us about violations of four different levels of MI (Widaman & Reise, 1997). These levels are configural invariance (invariance of the number of factors and the pattern of non-zero loadings), weak invariance (invariance of the non-zero loadings), strong invariance (invariance of the intercepts), and strict invariance (invariance of the unique variances). Strict invariance is assumed to hold within each state, since the states capture differences in loadings, intercepts, and unique variances.

For obtaining the state-specific MMs, LMFA uses exploratory FA (EFA) and not confirmatory FA (CFA). CFA is too restrictive because it imposes a priori assumptions about the presence or absence of item–factor relations by setting certain loadings equal to zero. Thus, CFA cannot detect MM differences pertaining to the configural model, such as the number and nature of the underlying factors in our previous adolescent example. Note, however, that the EFA model is not identified without setting constraints. Firstly, one needs to set the scale of the factors. To this end, *lmfa* sets the factor (co)variances equal to an identity matrix (with dimensions equal to the state-specific number of factors), which means that factors are initially uncorrelated. This initial solution is usually not well interpretable because many items may have high loadings on more than one factor (i.e., there is no “simple structure”; Thurstone, 1947). In order to achieve a more interpretable solution, *lmfa* applies a rotation of the factors for each state. An oblique rotation (i.e., one that allows factors to be correlated) results in the best simple structure and is usually more valid for psychological constructs (Clarkson & Jennrich, 1988; De Roover & Vermunt, 2019; Kiers, 1997). Finally, the factor means are set equal to zero per state. This implies that the state-specific intercepts are state-specific item means.

### The transition model

After examining the MMs, the next step is to investigate what the transitions between the MMs look like with the CT-LMM. As previously stated, the CT-LMM is a latent class model that allows subjects to transition between latent states over time. Specifically, we will inspect the probability of starting in a state at the first time point (i.e., “initial state probabilities”) and the probabilities of transitioning to other states from one time point to the next (i.e., “transition probabilities”).

#### Initial state parameters

The initial state probabilities pertain to the probability of starting in a particular state at the first time point. The probabilities sum to 1 and are stored in a vector with elements equal to the number of states. For example, the vector **π** = (.42 .34 .24) shows that the probability of starting in state 1 is .42, the probability of starting in state 2 is .34, and the probability of starting in state 3 is .24. In *lmfa*, logit models are used to model the initial state probabilities (similar to logistic regression; Agresti, 1990). The inherent logit values (or “log-odds”) indicate the relative chance of starting in a state compared to a reference state (in *lmfa*, this is state 1). Note that a separate logit model is required for all states but the reference state. These logit values do not have to be interpreted, because the initial state probabilities can be calculated from these logit models (see Supplementary Material S.2.1).^4^

Finally, the initial state parameters may be related to covariates, such as scores on a baseline questionnaire (e.g., a depression score or a score for the general ability to differentiate between emotions). However, including covariates on the initial state parameters only makes sense if the dataset contains data of more than a few subjects.^5^ Otherwise, there is not enough information to investigate the covariate effects. The covariates are related to the initial state parameters through regression, and they affect the logits and not the probabilities directly (see Supplementary Material S.2.1). However, to see the covariate effects on the initial state probabilities, one can convert logits into probabilities for different covariate values and compare them. For example, for a categorical covariate with two categories, one could compare the initial state probabilities for both categories. For continuous covariates, one could compare the initial state probabilities corresponding to the sample mean plus one standard deviation of the covariate to the probabilities corresponding to the sample mean minus one standard deviation (or compare probabilities for different quantiles of the covariate) while setting other covariates equal to their (sample) means.

#### Transition parameters

The transition probabilities are stored in a matrix with dimensions equal to the number of states, and the elements within a row of the transition probability matrix sum to 1 (Bartolucci et al., 2015; Zucchini et al., 2016). To clarify this, consider the following matrix:

1 $$\mathbf{P}=\left(\begin{array}{ccc}{p}_{11}=.66& {p}_{12}=.18& {p}_{13}=.16\\ {}{p}_{21}=.20& {p}_{22}=.49& {p}_{23}=.31\\ {}{p}_{31}=.32& {p}_{32}=.17& {p}_{33}=.51\end{array}\right).$$

The rows indicate the state memberships at the previous time point, and the columns indicate the current ones. Thus, the diagonal values specify the probabilities of staying in a state, and the off-diagonal elements refer to the probabilities of transitioning to another state. For instance, the first row of the matrix shows that the probability of staying in state 1 is equal to .66, and the probabilities of transitioning from state 1 to state 2 and from state 1 to state 3 are equal to .18 and .16, respectively.

As described before, the transition probabilities depend on the interval between two consecutive measurement occasions. The larger the interval, the larger the probabilities of transitioning to another state.^6^ To accommodate the interval length, LMFA (using CT-LMM) does not estimate the transition probabilities directly. Instead, transition intensities (or “rates”; i.e., transition probabilities per very small time unit)^7^ are estimated, and the transition probabilities are computed based on the transition intensities and the intervals (Böckenholt, 2005; Jackson & Sharples, 2002).^8^ The transition intensities are also captured in a matrix with dimensions equal to the number of states. However, intensities are only estimated for the transitions away from the origin state and, hence, for the off-diagonal entries. The diagonal entries equal the negative sum of the off-diagonal transition intensities, implying that rows sum to zero (Cox & Miller, 1965). For example, consider the matrix that corresponds to the transition probabilities in Eq. (1):

2 $$\mathbf{Q}=\left(\begin{array}{ccc}-{q}_{12}-{q}_{13}=-.51& {q}_{12}=.31& {q}_{13}=.20\\ {}{q}_{21}=.20& -{q}_{21}-{q}_{23}=-.86& {q}_{23}=.66\\ {}{q}_{31}=.56& {q}_{32}=.28& -{q}_{31}-{q}_{32}=-.84\end{array}\right).$$

The rate to transition from state 1 to state 2 is *q*_12_ = .31, and the rate to transition from state 1 to state 3 is *q*_13_ = .20. Larger rates are related to larger transition probabilities away from a state.

The transition intensities are modeled through a log-linear model such that the parameters are not intensities but log intensities (thus, the parameterization differs from the logit parameterization of the initial state parameters). For example, the estimates for the log intensities corresponding to intensities for the first row in Eq. (2) are log(*q*_12_ = .31) =  − 1.17 and log(*q*_13_ = .20) =  − 1.60. Intensities can be obtained from the log intensities by exponentiation (e.g., e^−1.60^ = .20).

Finally, like the initial state parameters, the transition parameters may be related to covariates, which may be time-constant, such as scores from baseline questionnaires, or time-varying^9^, such as the negative event scores and the intervention some subjects receive during their participation in our example data. The covariates are related to the transition parameters through regression (as is the case for the initial state parameters; see Supplementary Material S.2.1). Because the parameters of the transition model are log intensities, the regression effects have to be exponentiated to obtain the effects of the covariates on the transition intensities. However, it is more convenient to interpret the covariate effects on the transition probabilities. To this end, one can convert the intensities into probabilities for a certain interval length and different covariate values and compare them (as for the initial state probabilities).

### Estimation

In *lmfa,* the maximum likelihood (ML) parameter estimates are obtained with the 3S estimation (Vogelsmeier et al., 2021a), which builds on Vermunt's (2010) ML method and its extension for DT-LMM by Di Mari et al. (2016). The 3S estimation separates the estimation of the state-specific MMs and the CT-LMM as follows:

1. The state-specific MMs are estimated while disregarding the transitions between the latent states at consecutive measurement occasions and the covariate effects on these transitions.
2. Each observation is assigned to the state with the highest state-membership probability; that is, “modal state assignment” is applied.^10^ Furthermore, the inherent classification uncertainty is calculated. Note that there is always uncertainty unless all observations are assigned to a state with a probability of 1.
3. The MMs (i.e., the factor parameters) are kept fixed, and the state assignments from step 2 are used as single indicators to estimate the CT-LMM (with covariates) while correcting for step 2’s assignment uncertainty. This correction is necessary to prevent underestimating the relations between the states (i.e., the transition probabilities) and the covariate effects. Also, note that the final state assignments will differ slightly from the step 2 state assignments (see Supplementary Material S.4 and S.5). Usually, the assignments improve because the step 3 estimation benefits from additional information from the transition model (with covariates) to classify the observations (Vogelsmeier et al., 2021a).

For technical details about the steps, their likelihood functions, and the algorithms to maximize them, see Supplementary Material S.3–S.5.

## How to conduct LMFA with the *lmfa* package

**Fig. 1:**
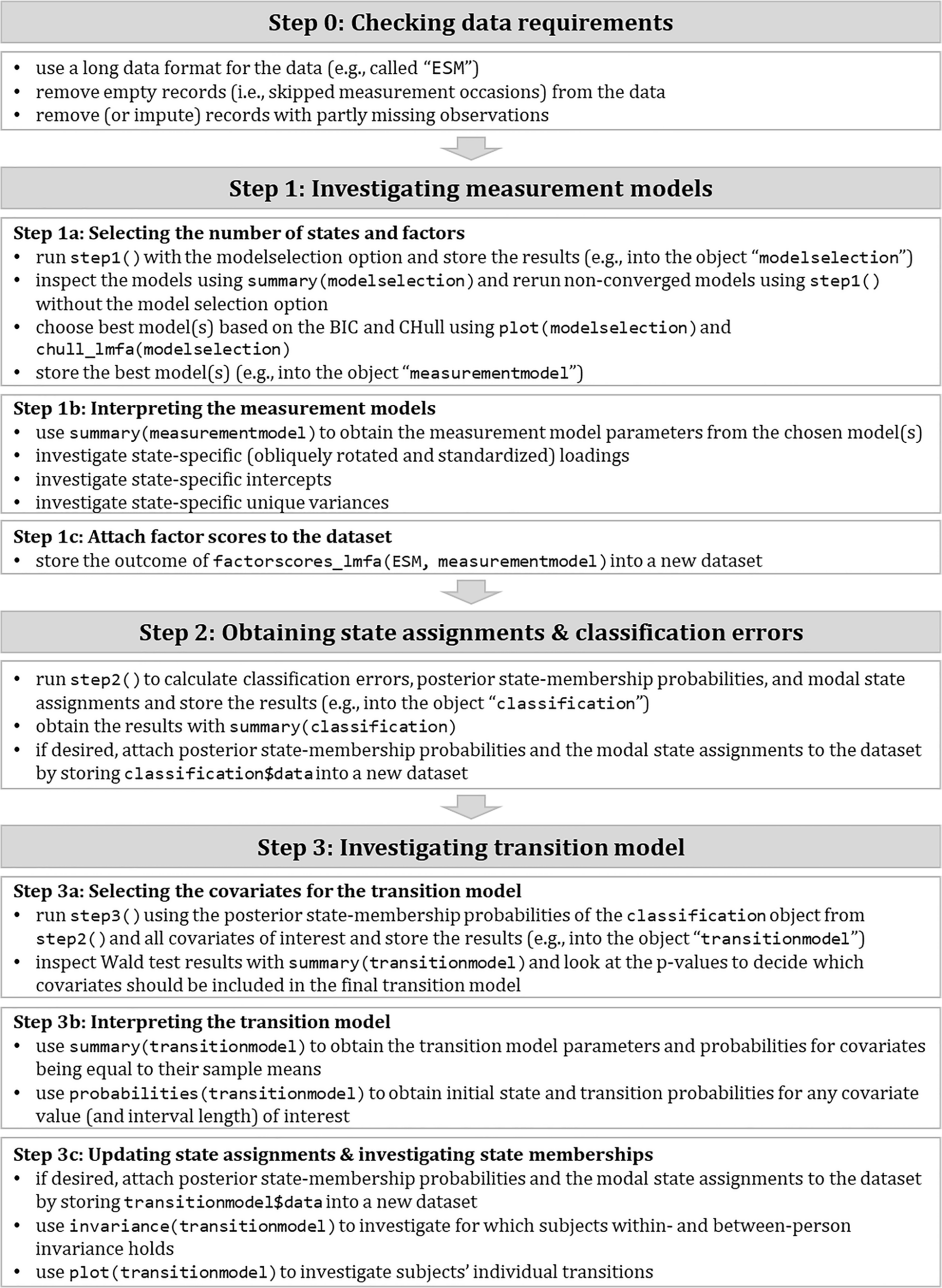
Summary of the three steps to conduct latent Markov factor analysis with *lmfa*.

In the following, we guide the reader through the different steps of conducting LMFA in the package *lmfa*. These steps are based on the three estimation steps described in “Estimation” section: Step 1 is investigating the MMs, step 2 is obtaining the state assignments and classification errors, and step 3 is investigating the transition model. Note that we introduce an additional step 0, which pertains to checking the data requirements prior to performing LMFA. Furthermore, as mentioned in “Illustrative example” section, the best model complexity in terms of the number of states and factors is unknown in advance and has to be evaluated in step 1. Additionally, depending on the subsequent analyses to investigate dynamics in psychological construct, researchers require factor scores corresponding to the state-specific MMs. Therefore, step 1 is divided into selecting the number of states and factors (step 1a), interpreting the MMs (step 1b), and attaching factor scores to the dataset (step 1c). Moreover, one must decide which covariates to include in the final transition model. Additionally, as mentioned in “Estimation” section, the state assignments should be updated after estimating the transition model and before inspecting subjects’ final state memberships. Therefore, step 3 is divided into selecting covariates (step 3a), interpreting the transition model (step 3b), and updating the final state assignments and investigating the state memberships (step 3c). Figure 1 summarizes the steps with references to the required *lmfa* functions.^11^

In this section, we describe the steps and functions using our example data introduced in “Illustrative example” section. In order to follow the steps of this tutorial, the *lmfa* package and the example data have to be loaded into R. Before using the package for the first time, it must be installed once. This can be done using the following command:

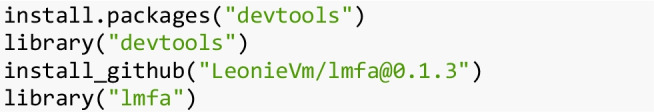



Note that the package *devtools* is required to install packages from the GitHub repository. The dataset used in this tutorial can be loaded into the R environment with the command:

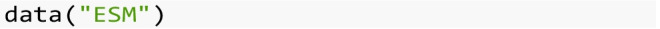



### Step 0: Checking data requirements

The first step is to check the data requirements with regard to the format (“Data format” section) and missing values (“Missing data” section).

#### Data format

In line with the assumed data structure, the dataset must be in long format, with rows equal to the number of total observations. Furthermore, next to the columns with the indicators of the latent factors (in our case, ) and possibly covariates (in our case,  ), the data must contain a column with the subject identification numbers (in our case,  ). Moreover, if observations should not be treated as equidistant, the user must specify a column with the time intervals between two consecutive observations (in our case,  ). Note that, prior to computing the intervals, rows corresponding to measurement occasions skipped by participants must be removed from the data (also see “Missing data” section) such that the intervals represent the time between *observed* measurement occasions. Additionally, a proper unit should be used. For instance, if there is approximately only one observation per day, the unit “days” is appropriate (e.g., with an interval of 1.42 days representing one day and 10 hours). If there are several observations per day, say nine, “hours” is an appropriate unit. With “minutes” or “seconds” as a unit, the intervals for these examples would take large values that likely lead to numerical problems when estimating the model.^12^ Furthermore, observations within subjects must be ordered by time (i.e., intervals must not be negative). Additionally, intervals for consecutive observations within a subject must not be equal to zero. Zero and negative intervals may occur from technical errors during data collection and should be corrected (otherwise, an error message is displayed).

#### Missing data

The data should include only records for the measurement occasions at which the subjects completed the questionnaires, because the CT-LMM automatically accounts for differences in the intervals, including skipped measurement occasions. More specifically, when excluding rows with skipped measurement occasions before computing the intervals (see “Data format” section), intervals between certain *observed* measurement occasions simply increase. Note that, depending on the data collection software or technical errors, it may happen that a subject started a questionnaire but did not finish it, such that some indicators or covariates contain missing values. This missing data should be dealt with before running *lmfa* (otherwise, an error message is displayed because the package cannot handle missing data yet). Cases with missing indicators should be removed.^13^ For cases with only missing covariates, imputation may be applied (e.g., using the single stochastic regression imputation in the *mice* package in R; van Buuren & Groothuis-Oudshoorn, 2011). Note that removing and imputing missing data can impact the validity of the findings (Lang & Little, 2018). For instance, single imputed values may lead to underestimation of the standard errors and thus to inflated type 1 errors when evaluating covariate effects in step 3 of LMFA. Therefore, results should be interpreted with caution.

### Step 1a: Selecting the number of states and factors

When estimating an LMFA model, the number of underlying states and factors per state must be specified. For our example data (see “Illustrative example” section), the data-generating model was one with three states and three factors in the first and third states and two factors in the second state. However, when analyzing real data with an exploratory approach like LMFA, the best model complexity is not known in advance. It must be determined by estimating several plausible models and comparing their results in terms of fit and parsimony. To this end, one can use criteria that balance the loglikelihood and number of parameters, such as the Bayesian information criterion (BIC; Schwarz, 1978) and the convex hull (CHull; Ceulemans & Kiers, 2006) method (Bulteel et al., 2013; Vogelsmeier et al., 2019b).^14^ In the following, we first describe the two criteria (“BIC and CHull” section). Then, we explain how to determine what range of states and factors to include in the model selection procedure (“Range of states and factors” section) and how to increase the chance of finding the “global” maximum and how to assess convergence of the estimation procedure (“Increasing the chance to find the global maximum and assessing convergence” section). Finally, we show how to perform the model selection with *lmfa* (“Model selection with *lmfa*” section).

#### BIC and CHull

First, the BIC considers model fit and complexity by penalizing models with a larger number of parameters (see Supplementary Material S.6.2). Second, the CHull is a generalized scree test (Bulteel et al., 2013; Ceulemans & Kiers, 2006) that automatically identifies models at the higher boundary of the “convex hull” (or CHull) in a “loglikelihood vs. number of parameters” plot (Cattell, 1966) and that chooses the best model by finding the point (or “elbow”) in this scree plot (or CHull plot) at which improvement in fit levels off when adding additional parameters to the model. Detecting this elbow is done by comparing “scree ratios” (see Supplementary Material S.6.3) for all models on the upper boundary, and the model with the largest ratio is chosen. In this way, the CHull also balances complexity and parsimony.

Both the BIC and the CHull offer valuable information about which model should be selected. However, for many real datasets, the BIC may keep decreasing when adding additional states and/or factors to the model (Bauer, 2007; McNeish & Harring, 2017). Then, investigating the relative improvement in the loglikelihood value using the CHull is especially important. Additionally, the CHull does not make distributional assumptions and may therefore perform better for many empirical datasets. However, the CHull method has two drawbacks that should be accounted for. First, the least and the most complex models at the higher boundary of the CHull cannot be chosen because no scree ratios can be computed (see Bulteel et al., 2013). Therefore, it is always advisable to inspect the CHull plot visually (e.g., the most complex model might still fit considerably better than the preceding model on the hull). The *lmfa* package will remind the user of this by displaying a note. Second, the scree ratio may be artificially inflated in some cases, even though the more complex model does not add much in terms of the fit. Specifically, when adding additional parameters hardly increases the fit anymore, both the numerator and denominator of the scree test ratio (Supplementary Material S.6.3) approach zero, which results in a very large scree test ratio, whereas the hull is pretty much a straight and horizontal line at that point (for a detailed explanation, see Wilderjans et al., 2013). The *lmfa* package displays a message if there are signs of artificial inflation. When the message is displayed, the user should inspect the CHull plot visually and also consider the next-best model(s). Finally, it is best practice to look at the results of competing models and take the interpretability into account.

#### Range of states and factors

For the model selection, one must decide on the range of states and factors. Regarding the former, one may start with a few states (say, 1–3). If models with three states barely improve model fit (i.e., according to the BIC and CHull) or if the estimation of three states already causes estimation problems, there is no point in adding more states. Moreover, the maximum number of states is restricted by the number of observations (i.e., one should have at least 1000 observations for each state; Vogelsmeier et al., 2019b). For instance, we should not include more than four states for our example dataset (with 4776 observations). To decide on the number of factors, one should think about theoretically plausible factor structures and consider that each factor should ideally be measured by at least three items. Otherwise, the factors may not be well measured or “determined”, which may cause convergence problems, Heywood cases (Van Driel, 1978), or less reliable parameter estimates. For example, suppose the data consist of six indicators, of which three are intended to measure positive affect, and three are intended to measure negative affect. In that case, no more than two factors should be included. Additionally, similarly to the number of states, one should begin with a few factors and examine the increase in fit and convergence problems for the most complex factor structure.

#### Increasing the chance of finding the global maximum and assessing convergence

For estimating the state-specific FA models, the algorithm searches for the maximum of the loglikelihood function (Supplementary Material S.3), that is, the solution with the largest loglikelihood value. However, it is possible that the solution is not a “global” maximum but a “local” one. To clarify this, consider the loglikelihood function as a landscape with multiple hills. Each hill has its own local maximum (i.e., the top), but only one hill is the highest and thus has the global maximum. In order to start searching for a global or local maximum, the algorithm requires initial parameter values. Different start values may lead to finding different (local) maxima (comparable to searching for the highest hill starting from different locations in the landscape). Therefore, the algorithm needs to use multiple start sets with different initial values and, in the end, provide the solution with the best loglikelihood value (Supplementary Material S.3.5). Users should choose at least 25 start sets, but the larger the number of start sets, the more likely it is to obtain the solution pertaining to the global maximum.

Moreover, it is possible that the model estimation does not converge at all. This means that the algorithm did not find a (local or global) maximum in a prespecified number of maximum iterations (Supplementary Material S.3). Especially for more complex models, the algorithm may require more iterations to achieve convergence. However, it could also signify that the model is not suited for the data (e.g., too many factors). The user may decide to re-estimate corresponding models once (and allow for more iterations) before continuing with the model selection procedure. The *lmfa* package displays this advice as a reminder.

#### Model selection with *lmfa*

To select the “best” model among the models with different numbers of states and factors, we have to use *lmfa*’s step1() function. To refer to the models, we use square-bracket labels. For example, the model from which example data have been generated (see “Illustrative example” section) is [3 2 3]. The number of elements inside the brackets is equal to the number of states. The first value in the brackets refers to the number of factors in the first state, the second value refers to the number of factors in the second state, and so on. Thus, model [3 2 3] refers to a model with three states and three factors in state 1, two factors in state 2, and three factors in state 3. In the following, we compare models with one to four states and one to three factors per state (i.e., 14 models in total; the models are displayed in LMFA output box 1). It is important to note that the state labels can switch in any mixture model when repeating the analysis. Thus, the labels are random across analyses. For example [3 2 3], [3 3 2], and [2 3 3] are different permutations of the same model (i.e., model [3 2 3] is the same as model [3 3 2] and [2 3 3]). Therefore, only one permutation of this model is estimated (and shown in the output). The function step1() can be used as follows (because the estimations start from random state-membership assignments (see Supplementary Material S.3.5), we set a seed for reproducibility):

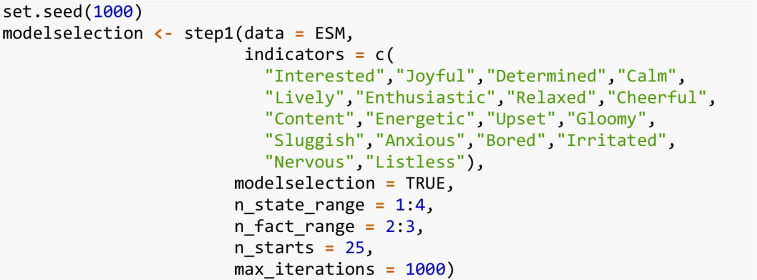



There are five mandatory arguments that we have to specify. First, we have to provide the data via the data argument (in our case, ESM). Second, via the indicators argument, we specify the variable names of the indicators in the same order as they appear in the data. These are  . Third, we indicate that we want to perform model selection via the argument  . Fourth and fifth, we determine the range of states and factors that should be included in the model selection with  and  . Additionally, we could change the default values for the number of start sets and the number of maximum iterations after which the estimation terminates regardless of whether convergence has been reached, but we simply use the default values  and  .^15^

When the estimation is terminated, we obtain the model-selection results as follows:

summary(modelselection)

Note that the model selection for our example data took about three hours. To follow the next tutorial steps in R, readers can simply load the model selection object with the command:  . The output is displayed in LMFA output box 1.

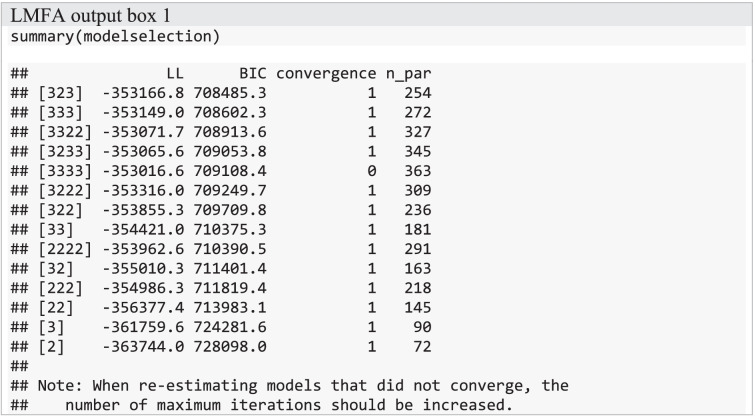



The first column (i.e., “LL”) pertains to the loglikelihood value. The second column (i.e., “BIC”) shows the value of the BIC. The third column (i.e., “convergence”) indicates whether the model estimation converged (with 1 = “convergence” and 0 = “non-convergence”). The fourth column (i.e., “n_par”) shows the total number of parameters.^16^ The models are ordered by the value of the BIC, starting with the lowest value and thus the model with the best fit according to this criterion. As described above, the state labels are random across analyses. However, *lmfa* provides consistency by reordering the states based on the size of the states, starting with the largest (i.e., the one with the most observations). For example, the order of model [3 2 3] in the first row of the LMFA output box 1 reveals that the largest state of this model has three factors, the second-largest one has two factors, and the smallest state has three factors.

Before continuing with the model selection, we check whether models have to be re-estimated due to non-convergence. Indeed, the estimation of model [3 3 3 3] did not converge. For estimating single models, we use the step1() function but without model selection (i.e., with  ). The code to estimate model [3 3 3 3] is:

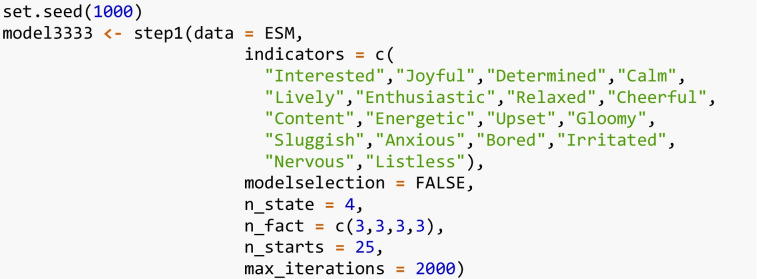



When  , it is mandatory to provide a single number of states via the argument n_state (i.e.,  ) and a vector with state-specific numbers of factors via the argument n_fact (i.e.,  ). As previously described, when re-estimating models that initially did not converge, it is wise to increase the number of maximum iterations. Therefore, we set  . We replace the old models by the new models with:

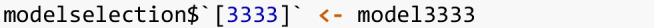



However, the model did not converge (it might simply not be suitable for the data), and therefore we continue with the original model selection object.

From the summary in LMFA output box 1, we can see that the best model according to the BIC is model [3 2 3] and, thus, the data-generating model. For an easier inspection of the results, we also plot the BIC of the converged models against the number of free parameters:

plot(modelselection)

The output is shown in LMFA output box 2.

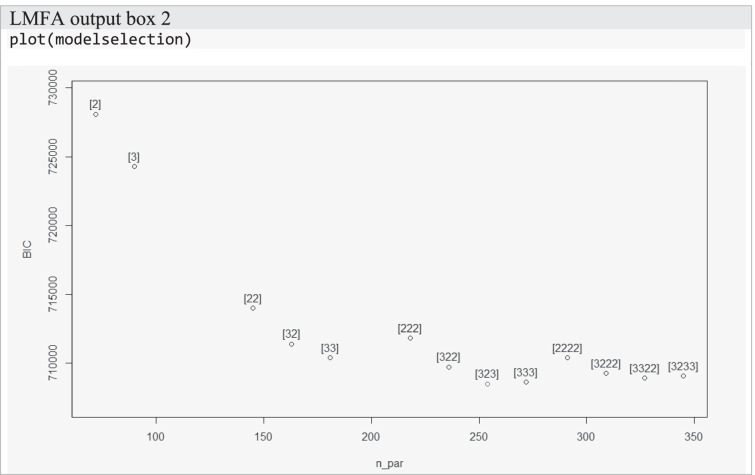



A red dot indicates the model corresponding to the lowest BIC value. Note that, for our example, the BIC does not keep increasing for more complex models. Therefore, we would consider it relatively safe to choose the model with the lowest BIC value. However, to support our choice, we also investigate the results of the CHull method for the converged models, which can be obtained with the chull_lmfa() function as follows:

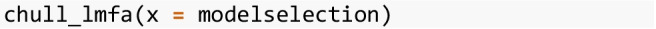



We only have to specify argument x, which pertains to the model-selection object (in our case, modelselection).^17^ The output is shown in LMFA output box 3.

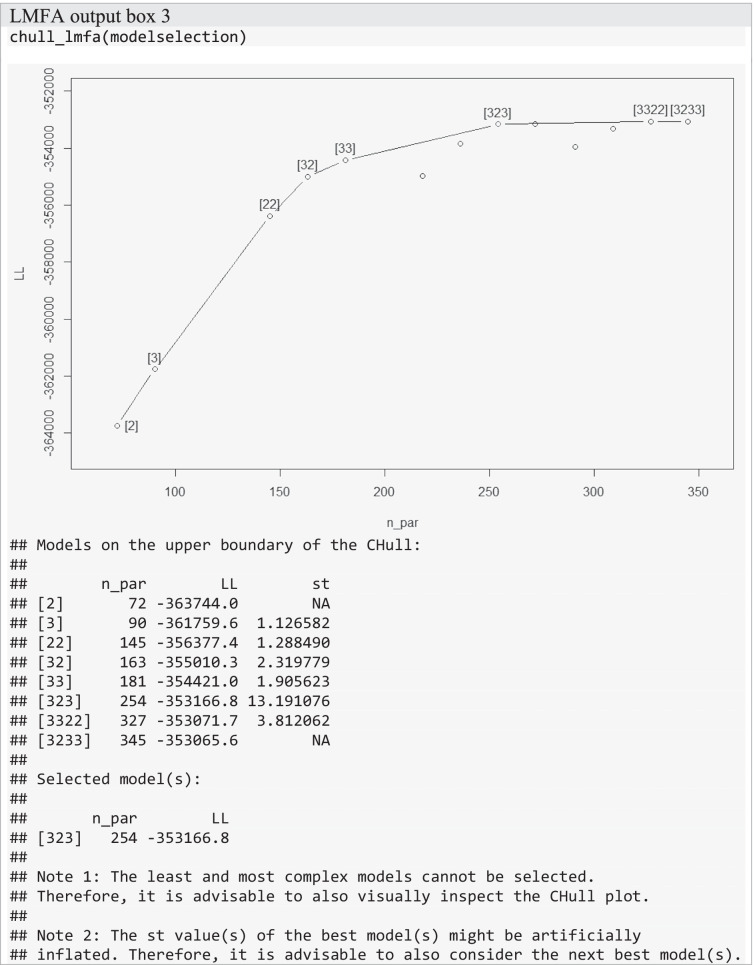



The output consists of three parts, the CHull plot, the summary of the models on the upper boundary of the CHull (including their scree-test values “st”), and the selected model(s). We see that the model [3 2 3] was selected. However, we received the note that the scree value might be artificially inflated. Therefore, one should also consider the results of the next-best model(s). Because we know the data-generating model, we continue with model [3 2 3].^18^ To inspect the model, we have to extract it from the model-selection object modelselection and store it as follows:

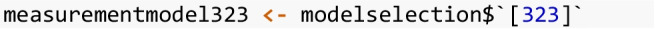



The parameters can be displayed with the command:^19^^,^ 20

summary(measurementmodel323)

### Step 1b: Interpreting the measurement models

After selecting the model, we can interpret the MMs shown in LMFA output box 4.^21^

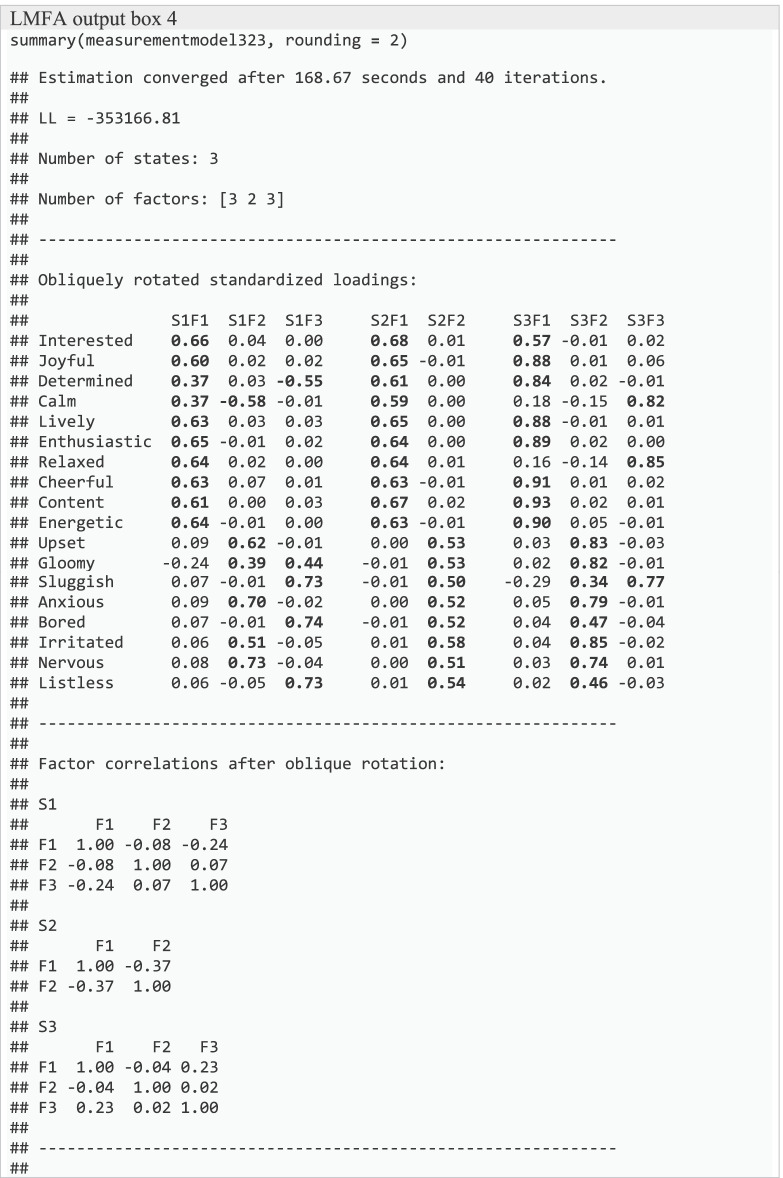

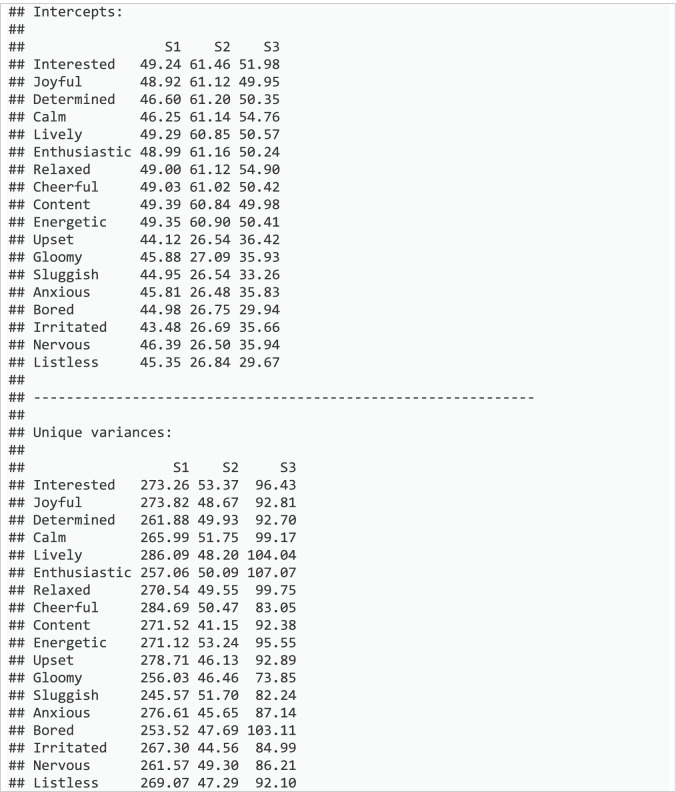





#### Intercepts

We first look at the intercepts for this data example because it allows us to give labels to the states that we use throughout the interpretation of the other parameters. Specifically, we see that the intercepts for the positive emotions (i.e., “interested”, “joyful”, “determined”, etc.) are larger than the intercepts for the negative emotions (i.e., “upset”, “gloomy”, “sluggish”, etc.) in each of the three states (i.e., “S1”, “S2”, and “S3” in the output). However, intercepts differ across states such that the intercepts for the positive emotions are largest in state 2, followed by state 3 and then state 1, and the intercepts for the negative emotions are largest in state 1, followed by state 3 and then state 2. Therefore, in the following, we label the first state the “displeasure” state, the second one the “pleasure” state, and the third one the “neutral” state.

#### Loadings

Next, we inspect the loadings. Note that the default output displays standardized^22^ obliquely rotated factor loadings.^23^ The reason is that unstandardized values can be difficult to interpret, as they often exceed an absolute value of 1 (especially when a large rating scale is used like in our example dataset; “Illustrative example” section), and hence, rules of thumb to evaluate which items have strong loadings on a factor cannot be applied. In contrast, for standardized loadings, rules of thumb are available (e.g., loadings with an absolute value larger than or equal to 0.3 can be seen as considerable, which is also the threshold used in our example).^24^

Looking at the loadings, we see that, in all states, the first factors (i.e., “S1F1”, “S2F1”, and “S3F1” in the output) correspond to a “positive affect” (PA) factor containing loadings (≥ 0.3) of most or all positive emotion items. However, for the first factor in the displeasure state (“S1F1”), the loadings of the items “determined” and “calm”—both equal to 0.37—are somewhat lower than the loadings of the other positive emotions on this factor. Furthermore, for the first factor in the neutral state (“S3F1”), the loadings “calm” and “relaxed”—equal to 0.18 and 0.16—are even lower than the chosen threshold of 0.3. Furthermore, the second factors (i.e., “S1F2”, “S2F2”, and “S3F2”) mainly have large loadings for negative emotions but with differences across states. Specifically, while the pleasure state has an apparent "negative affect” (NA) factor (“S2F2”) with high loadings of all negative emotions, the displeasure state has a bipolar “distress” factor (“S1F2”) with loadings of mainly high-arousal negative emotions (i.e., “upset”, “gloomy”, “anxious”, “irritated”, and “nervous”) and a reversed loading of the item “calm”. The second factor in the neutral state (“S3F2”) has characteristics of the second factors of both the pleasure state (“S2F2”) and the displeasure state (“S1F2”) in that it has considerable loadings of all the items but relatively low loadings of the low-arousal emotions (i.e., “sluggish”, “bored”, and “listless”). The most striking difference is that the displeasure state contains a third bipolar “drive” factor (“S1F3”), whereas the neutral state contains a third “serenity” factor (“S3F3”). More specifically, the drive factor (or rather lack-of-drive factor; “S1F3”) has high loadings of the low-arousal negative emotions “gloomy”, “sluggish”, “bored”, and “listless” and a reversed loading of the item “determined”. The serenity factor (“S3F3”) has high loadings of the low-arousal emotions “calm”, “relaxed”, and “sluggish”. In conclusion, subjects in the displeasure or neutral state have a more differentiated representation of their emotions than in the pleasure state. The drive factor in the displeasure state aligns with research showing that drive differs from general positive affect when persons are anhedonic (Berridge et al., 2009; Treadway & Zald, 2011).

It is also interesting to inspect the factor correlations that result from the oblique rotations—which are not part of the MM. First, in the displeasure state (“S1”), we see a small negative correlation between PA (“F1”) and the lack-of-drive factor (“F3”). In the neutral state (“S3”), we see a small positive correlation between PA (“F1”) and the serenity factor (“F3”). In the pleasure state (“S3”), PA (“F1”) and NA (“F2”) are moderately negatively correlated. All other correlations are close to zero, indicating that the other factors are relatively independent of one another.

#### Unique variances

Finally, looking at the unique variances, we see that they are largest in the displeasure state (“S1”), followed by the neutral state (“S3”) and the pleasure state (“S3”). The large emotion-specific variability in the displeasure state is in line with findings that depression and emotional complexity are related (Grühn et al., 2013).

### Step 1c: Attach factor scores to the dataset

Before proceeding with step 2, we can attach state-specific factor scores to our dataset for each observation in the dataset.^25^ The factor scores are estimates of the latent constructs and can be used for subsequent analyses to investigate dynamics in psychological constructs (for suggestions on how to proceed in the presence of non-invariance, see “Proceeding based on the results of LMFA” section). A copy of the dataset with the factor scores attached can be obtained with:^26^

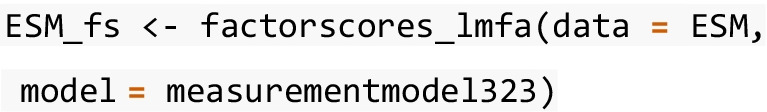



In this function, two arguments are required. First, via the argument data, we have to provide the data used for the step1() estimation (in our case, ESM). Second, via the argument model, we have to specify the step1() object with the state-specific MMs (in our case, measurementmodel323). In the resulting dataset (i.e., ESM_fs), the columns are called “S1F1”, “S1F2”, etc., where “S” refers to the state and “F” to the factor.

### Step 2: Obtaining state assignments and classification errors

The next step is to obtain information about the classification and the (modal) state assignments. In this section, we first describe how to obtain the results for our example data with *lmfa,* and then, based on the output, we explain the different classification statistics.

In order to obtain the classification information, we use the step2() function as follows:

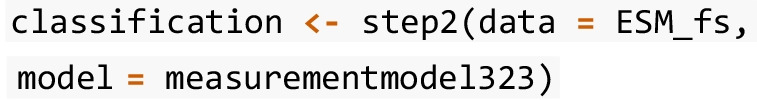



The function contains two arguments that we have to specify. First, we must provide the data used for the step1() estimation via the argument data. It is most convenient to use the version including the factor scores estimates (in our case, ESM_fs), because we will add additional columns later on and because this allows us to obtain a complete dataset for further analyses. Second, we need to specify the step1() object with the state-specific MMs via the argument model (in our case, measurementmodel323).^27^ The following code prints the results:^28^

summary(classification)

The output is shown in LMFA output box 5.

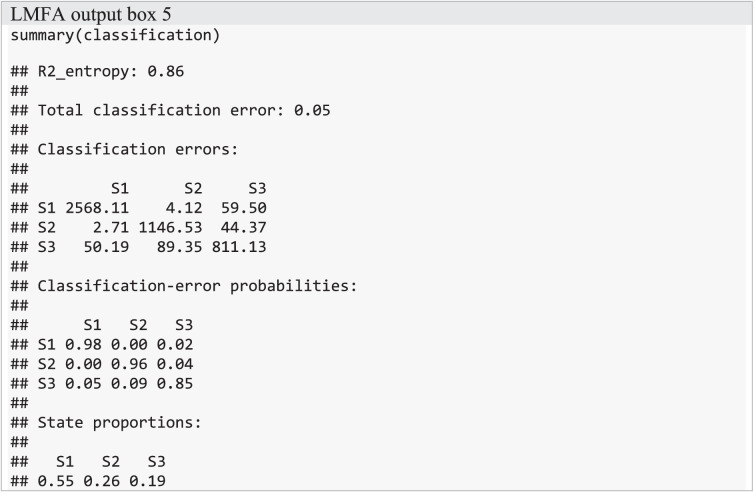



First, the R-squared measure ${R}_{entropy}^2$ (called “R2_entropy” in the output) indicates how well the states are separated (and thus how much the MMs differ), with values ranging from 0 (bad separation) to 1 (good separation; Lukočienė et al., 2010). Note that a larger state separation implies less classification error. It is important to inspect the ${R}_{entropy}^2$ value because a bad state separation (with ${R}_{entropy}^2<.5$) can lead to an incorrect classification error correction^29^ and, in turn, to an underestimation of transition probabilities and the covariate effects (Vermunt, 2010). When observing a bad state separation, which is rather unlikely in practice, it is advisable to use the FIML estimation, which is currently only available in LG. The ${R}_{entropy}^2$ value for our example data indicates that the states are well separated, which explains the small total classification error (called “Total classification error” in the output).

Second, information about the classification errors can be obtained from the classification error matrix (called “Classification errors” in the output), which cross-classifies the modal state assignments by the “true” state assignments and which is used to correct for the error in step 3 of the analysis (see Supplementary Material S.4). Higher values on the diagonal and lower values on the off-diagonal indicate minor classification error. For an easier interpretation, the counts can be translated into proportions (called “Classification-error probabilities” in the output). Inspecting the classification error matrices, we see that the error is lowest in the displeasure state (“S1”), followed by the pleasure state (“S2”) and the neutral state (“S3”). Thus, the classification into the neutral state was accompanied by the greatest uncertainty, which is not surprising as the neutral state is somewhat in between the displeasure and pleasure state.

Third, the state proportions (also called like that in the output) pertain to the state sizes. The displeasure state (“S1”) is largest, followed by the pleasure state (“S2”) and the neutral state (“S3”).

Finally, the state assignments are not displayed in the output because R cannot display all assignments simultaneously. However, we can simply obtain a copy of our dataset with additional columns corresponding to the state assignments with the following command:

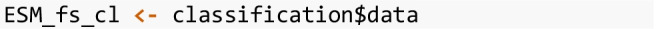



Specifically, the columns with the posterior state probabilities (in the dataset called “State1”, “State2”, etc.) indicate the probabilities for an observation to belong to a particular state and, thus, that the state-specific MM underlies the responses for this observation. As explained in “Estimation” section, the modal state assignments (called “Modal” in the dataset) correspond to the state with the largest probability and, hence, to the most likely state membership.

### Step 3a: Selecting the covariates for the transition model

When the state-specific MMs are obtained and the observations are assigned to the states, we can continue by investigating the transitions between the states and what may cause them by estimating an LMFA with covariates on the initial state and/or transition parameters. To test whether a covariate is significantly related to the transition model parameters (and, thus, whether it should be included in the model) Wald tests can be used (Agresti, 1990). In the following, we first explain the covariate selection with the Wald tests (“Covariate selection procedure using Wald tests” section) and then show how to perform the covariate selection for our example data with *lmfa* (“Covariate selection with *lmfa*” section).

#### Covariate selection procedure using Wald tests

Every covariate in the model is accompanied by separate covariate effects on the initial state or transition parameters (e.g., the covariate “had an intervention” has six effects, one on each of the transition parameters). The Wald tests in *lmfa* are chi-squared omnibus tests that show whether including a covariate is significant overall (i.e., across the initial and transition parameters). Thus, for every covariate, there is one Wald test statistic. To select which covariates to include, one can start with an LMFA with all covariate candidates. Then, the least significant covariate is removed, and the model is re-estimated. This backward selection continues until only significant covariates are left (say, according to an alpha level of .05). When only significant covariates are left in the model, one can continue to interpret covariate effects on the transition probabilities. Note that backward selection is only one possibility. Other covariate selection procedures may lead to selecting a different set of covariates (e.g., the forward selection; Heinze et al., 2018). Instead of using such a data-driven approach, a more theory-driven approach is also possible (e.g., investigating covariates that were significantly related to the transition model parameters in previous studies). In addition, model selection using Wald tests may be validated by comparing the BIC values of models that include different (sub)sets of covariates. However, a larger number of models must be estimated for this model comparison than when using the Wald tests (e.g., a model with all covariates included and excluded).

#### Covariate selection with *lmfa*

In the following, we estimate a transition model with covariate effects of “had an intervention” and “negative event” on the transition parameters. To estimate the transition model, we use the step3() function as follows (because the estimation starts from random values for the transition parameters (see Supplementary Material S.5.4), we set a seed for reproducibility):

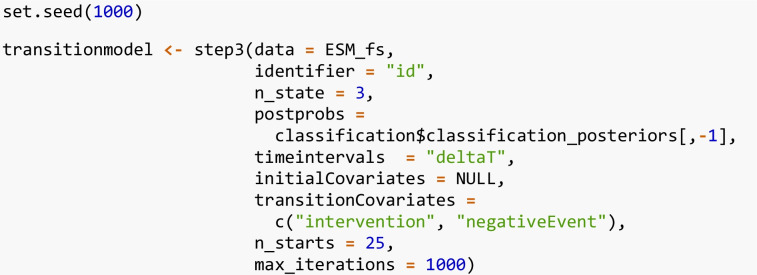



There are four mandatory arguments that we have to specify. First, we provide the data via the data argument. We use the dataset ESM_fs and, thus, the data including the factor scores but without the state assignments from step 2 because they are updated in step 3. Second, we specify the name of the column with the subject identification numbers via the argument identifier (in our case, ). Third, we define the number of states with  . Fourth, we specify the posterior state probabilities using the argument postprobs. The probabilities can be extracted from the step2() classification output with the command  , where  indicates that we leave out the column with the modal state assignments.

The following three arguments are not required but must be specified if the model should account for differences in intervals and if covariate effects on the transition model parameters should be included. Both apply to our example. Thus, first, via the argument timeintervals, we provide the function with the column's name in the dataset that contains the time intervals. In our case, this is  (if no such column name is provided, observations would be assumed to be equidistant). Next, via the arguments transitionCovariates and initialCovariates, we can provide (a vector of) column names that contain the covariate scores (the default for both arguments is NULL, i.e., no covariates are used). Thus, for our analysis, we provide the vector  as input for transitionCovariates.

Finally, similarly to the step1() function, the users may decide to change the default values for the number of start sets^30^ via the argument n_starts and the number of maximum iterations via the argument max_iterations^31^. However, we use the default values for our analysis, that is,  and  .^32^ After termination of the estimation, the results are obtained as follows^:^ 33

summary(transitionmodel)

The estimation for our example data took about 20 minutes. Again, readers who want to follow the rest of the tutorial can also load the results with the command:  . The results are presented in LMFA output box 6.

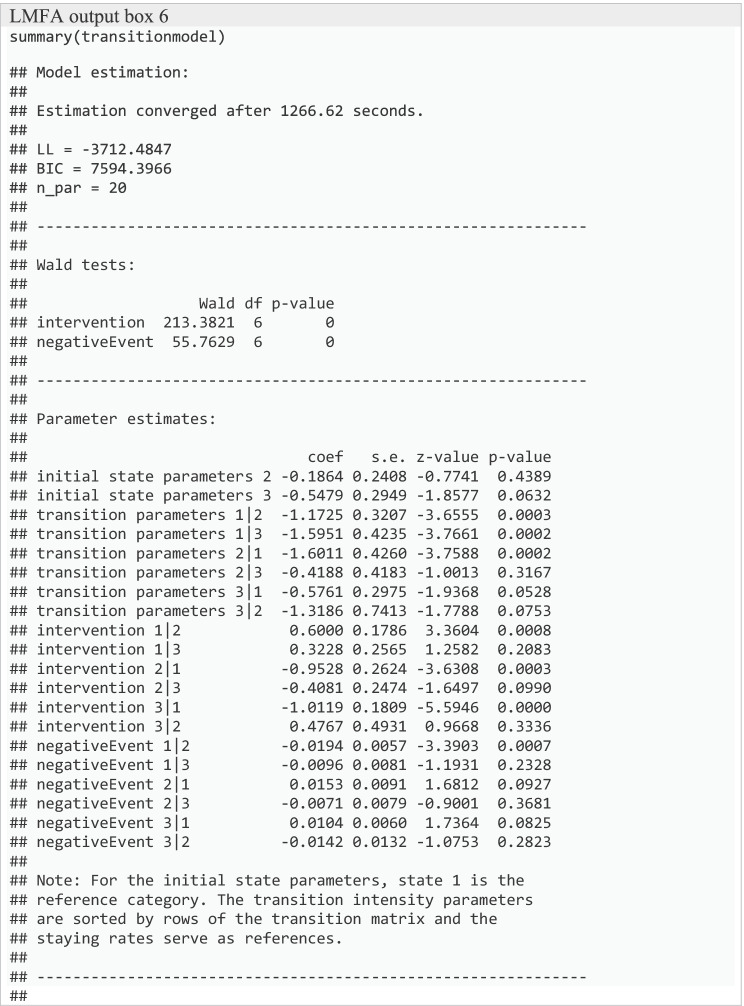

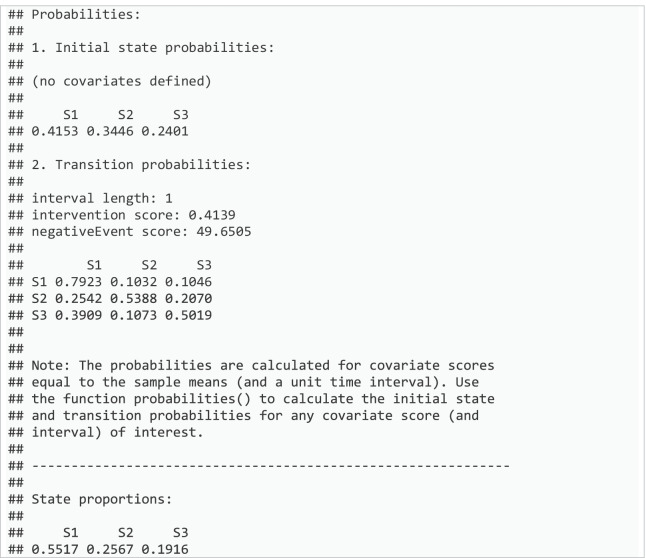





The results are shown in the “Wald tests” part in LMFA output box 6. For each covariate, we obtain a significance test with the corresponding Wald test statistic (i.e., “Wald”), the degrees of freedom^34^ (i.e., “df”), and the *p*-value (i.e., “p-value”). We see that both covariates have significant effects on the transition parameters. Thus, we keep both covariates in the model. The output also provides users with the BIC (also called like that in the output), including the number of parameters in the model (i.e., “n_par”). As described above, the BIC can be used to compare transition models with different (sub)sets of covariates. For our example data, the comparison of BIC values for transition models without covariates, with only one of the two covariates, and with both covariates resulted in the selection of the same model as when using the Wald tests alone and, therefore, will not be discussed further.

### Step 3b: Interpreting the transition model

After selecting the covariates for the transition model, we can interpret the effects on the probabilities and investigate changes in the state proportions. In the following, we first explain how to interpret (covariate effects on) the initial state and transition probabilities, then describe how to obtain additional insights by retrieving the initial and transition probabilities for any covariate values and time intervals of interest, and finally interpret changes in the state proportions.

#### Initial state probabilities

We first focus only on the parts “Parameter estimates” and “Initial state probabilities” in LMFA output box 6, starting with the former. The “coef” and “s.e.” columns indicate the point estimates and standard errors. The “z-value” and “p-value” columns show the corresponding *z*-statistics and two-tailed *p*-values. Since no covariates were included for the initial state parameters, there are only two “initial state parameters”. These parameters always correspond to the logit values for covariate scores equal to zero. In the case of covariate effects, they would be shown below the initial state parameters. As previously described, it is more convenient to interpret the corresponding initial state probabilities. More specifically, to obtain a good impression of what the probabilities look like for the average person, it makes the most sense to inspect the initial state probabilities for covariates being equal to the sample means. These probabilities can be found in the “Initial state probabilities” part further below in LMFA output box 6. Of course, if no covariates are defined (as in our model), the probabilities do not depend on the values of a covariate. The probabilities indicate that starting in the displeasure state (“S1”) was most likely, followed by the pleasure state (“S2”) and the neutral state (“S3”).

#### Transition probabilities

Next, we focus on the parts “Parameter estimates” and “Transition probabilities” in LMFA output box 6. The “transition parameters” in the “Parameter estimates” part correspond to the log intensities for covariate scores equal to zero. However, for better interpretability, we inspect the corresponding transition probabilities for a unit time interval for the average person in the sample, and thus, for covariate scores being equal to their sample means. These probabilities are displayed in the “Transition probabilities” part in LMFA output box 6. We can see that the sample mean for “had an intervention” is equal to .41, and the sample mean for “negative event” is equal to 49.65. The probabilities indicate that the probabilities of transitioning to another state are generally lower than staying in a state, especially when staying in the displeasure state (“S1”). The transition probabilities from the displeasure state (“S1”) to the pleasure state (“S2”) and the neutral state (“S3”) are approximately equal. The transition probability from the pleasure state (“S2”) to the neutral state (“S3”) is smaller than from the pleasure to the displeasure state (“S1”). Finally, the transition probabilities from the neutral state (“S3”) to the displeasure state (“S1”) are larger than the transitions to the pleasure state (“S2”).

#### Covariate- and interval-specific probabilities

To obtain the initial state and transition probabilities for any covariate score and interval of interest, we can use the probabilities() function. For example, to obtain the probabilities for a zero score on “had an intervention”, a “negative event” score equal to the sample mean of 49.65, and a unit interval, we use the following command:

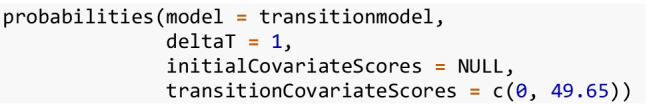



Only the first argument is mandatory; that is, we have to provide the output of the step3() function (in our case, transitionmodel) via the model argument. By default, the function prints the probabilities for a unit interval and covariate scores equal to the sample means (i.e.,  and  . To print the probabilities for specific covariate scores, we have to provide a vector with these scores in the same order as we included the covariates when estimating the transition model with the step3() function. In our case, we include  .^35^^,^ 36

For our example data, we make two comparisons. First, we set the “negative event” score equal to the sample mean and compare the probabilities for both categories of “had an intervention”. The probabilities for the “no intervention observations” are shown in LMFA output box 7.

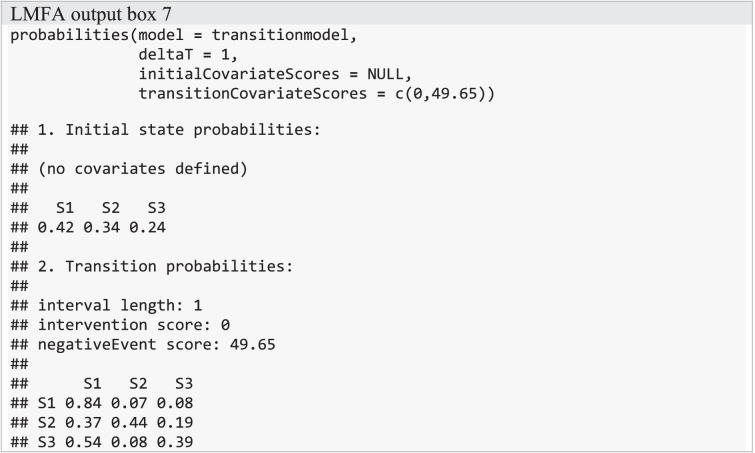



The probabilities for the “intervention observations” are displayed in LMFA output box 8.

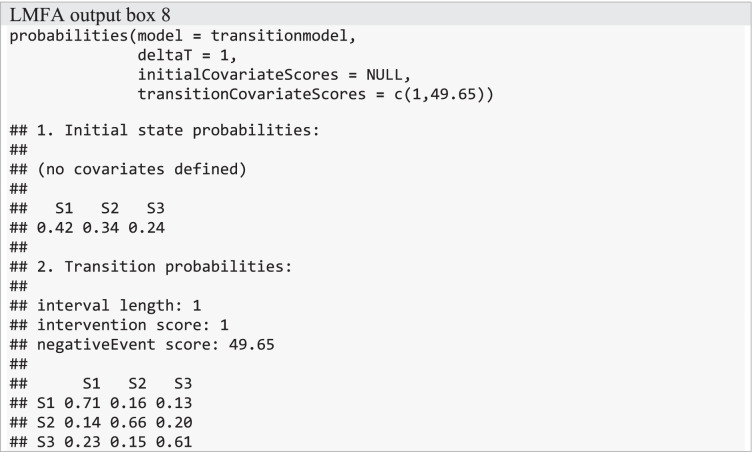



Comparing the transition probabilities, we see that having had an intervention is related to relatively smaller probabilities of transitioning to and staying in the displeasure state (“S1”).

Second, we compare the transition probabilities for the sample mean of “negative event” minus the standard deviation (i.e., 34.54) to the transition probabilities for the sample mean of the covariate plus the standard deviation (i.e., 64.76), thereby keeping the “had an intervention” score equal to the sample mean. The probabilities for a “negative event” score of 34.54 are displayed in LMFA output box 9.

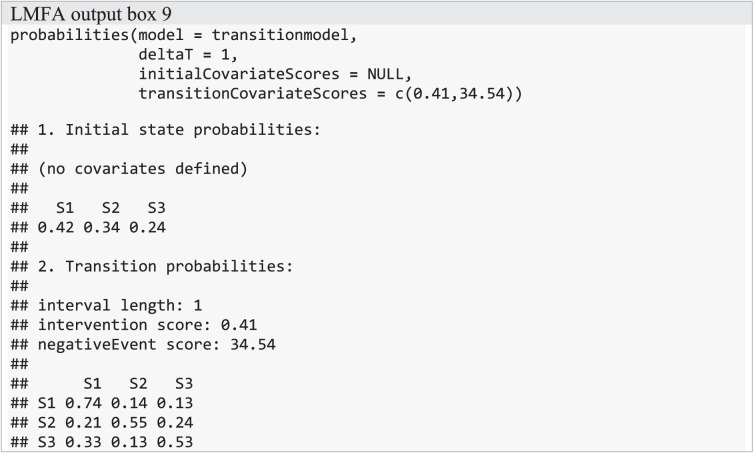



The probabilities for a “negative event” score of 64.76 are shown in LMFA output box 10.

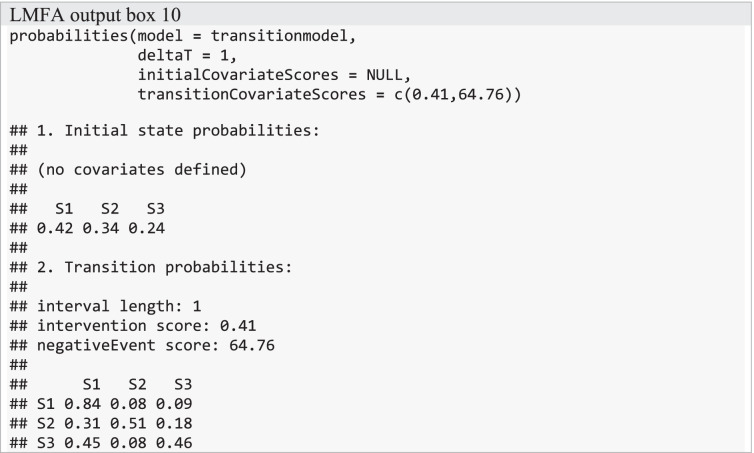



We see that higher scores on “negative event” are related to larger probabilities of transitioning to and staying in the displeasure state (“S1”).

#### State proportions

The final state proportions are shown under “State proportions” at the end of LMFA output box 6. We see no change when comparing the results to the state proportions in LMFA output box 5 (and thus to the state proportions resulting from the modal state assignment in step 2). This is not surprising considering the small classification errors from step 2.

### Step 3c: Updating state assignments & investigating state memberships

Next, we can again obtain a copy of our dataset with the final state assignments to see which observations are in which state and, thus, which observations are comparable:

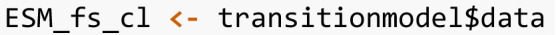



These state assignments should be considered for subsequent data analyses because, as described in “Estimation” section, they may be more accurate than the step 2 assignments. Therefore, we simply overwrite the previous dataset with the step 2 assignments ESM_fs_cl.

In addition to investigating which observations are invariant, it can be interesting to investigate which subjects have within-person invariance across their entire time of participation and for which subjects between-person invariance also holds in that they share the same permanent state. This information can be requested with the following command:

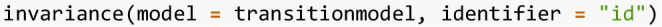



The function contains two arguments. First, we must provide the step3() estimation output via the argument model (in our case, transitionmodel). Second, we need to specify the name of the column with the subject identification numbers via the argument identifier (in our case, "id"). The output is displayed in LMFA output box 11.

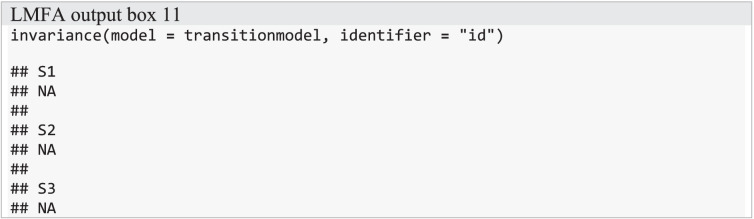



Subjects for whom within-person invariance holds are listed under the state that they are permanently in (i.e., either “S1”, “S2”, or “S3”). For subjects in the same permanent state, between-person invariance holds as well. However, in LMFA output box 11, we see “NA” (which stands for “Not Available”) for all three states, which means that within-person invariance does not hold for any of the subjects in the example data. Consequently, between-person invariance also does not hold for any of the subjects.

If one further wants to explore transitions of (some of) the subjects for whom within-person invariance is violated, one can request transition plots with the following command:

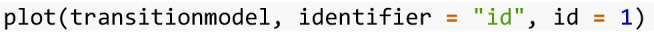



The function contains three arguments. Again, we have to provide the step3() estimation output via the argument model (in our case, transitionmodel) and the name of the column with the subject identification numbers via the argument identifier (in our case,  ). Moreover, we must specify the subject identification number for which we want to plot the transitions via the argument id (in our case,  ). LMFA output box 12 shows the transition plots for the first four subjects in the example data. As can be seen at the beginning of the output, the plots were obtained by looping over the id values1–4.

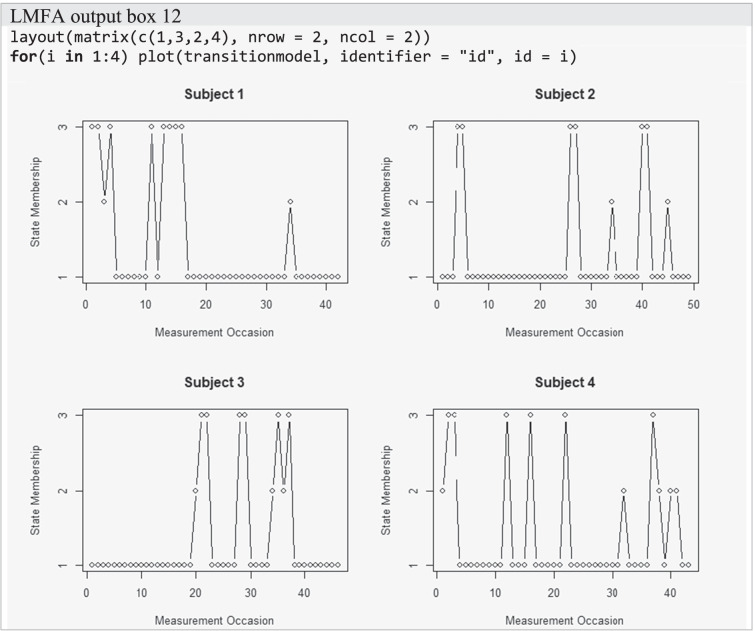



The *x*-axis shows the measurement occasions, and the *y*-axis shows the state membership corresponding to a particular measurement occasion. We see, for example, that all four subjects are in state 1 most of the time and that subjects 2 and 3 transition less often at the beginning of the study than at the end.

### Summary of the LMFA findings for our example data

In the following, we summarize the LMFA findings by answering the research questions posed in “Introduction” section.^37^

1. *How many MMs are underlying our ILD?* ➔Three MMs are underlying the example data.
2. *How do the MMs differ?* ➔The number and nature of the factors differ, implying that configural invariance is violated for our example data. More specifically, we found three states (a displeasure, a neutral, and a pleasure state) that all contained a positive affect and a negative affect (or distress) factor, but the displeasure state was additionally characterized by a drive factor and the neutral state by a serenity factor.
3. *How do subjects transition between the MMs over time, and is this related to time- or subject-specific covariates?* ➔Most subjects started in the displeasure state. The probabilities of staying in a state were generally higher than transitioning to another state (especially for subjects in the displeasure state). Transitions to the displeasure state were most likely, especially when experiencing negative events. After receiving an intervention, the probabilities of transitioning to and staying in the neutral or pleasure state increased.
4. *For which subjects does within-person invariance hold over time, and for which of these subjects does between-person invariance hold?* ➔Within-person invariance does not hold for any of the subjects and, therefore, neither does between-person invariance.

## Proceeding based on the results of LMFA

Once the MM differences and possible explanations are known, the question becomes how to proceed, based on the LMFA results, with (originally planned) analyses to investigate the dynamics in psychological constructs. The answer to this question largely depends on the findings. It is important to note that a comparison of the state-specific MMs may indicate violations of different levels of invariance and that the required level of invariance depends on the type of comparisons one wants to make. When comparing state-specific loadings, one may find that the MMs differ considerably across states—specifically, in the number and/or nature (the zero-loading pattern) of measured constructs—which indicates a violation of configural invariance. It may also be that the pattern of (near-)zero loadings appears to be equal across states but that the non-zero loadings differ in size. This suggests that configural invariance holds, but weak invariance fails. When configural or weak non-invariance is indicated, continuing with analyses that assume invariance is not possible for the entire dataset because factor scores are not validly comparable. Differences in the means of the constructs or relations between constructs could be due to underlying differences in the MMs. However, finding such differences in MMs is interesting in its own right (e.g., the additional drive factor for anhedonic subjects in our data example). In any case, it is possible to proceed with factor scores from one specific state (e.g., the largest state or the state that best corresponds to an a priori assumed MM) and, thus, with observations for which strict invariance holds.^38^

If weak invariance holds across the states—that is, if the (near-)zero and non-zero loadings are highly similar across states—users may examine whether covariances (e.g., regression coefficients or autocorrelations) between latent constructs (e.g., positive affect and negative affect) differ across subjects and/or change across time, because factor covariances are not affected by intercept differences (Oberski, 2017; Steenkamp & Baumgartner, 1998). However, examining whether mean construct scores differ across subjects and time points calls for strong invariance to avoid mixing up differences in latent means and intercept differences (Meredith & Teresi, 2006). This implies that strict invariance is not necessary for meaningfully comparing latent covariances or means (Putnick & Bornstein, 2016; Vandenberg & Lance, 2000). Thus, finding states that differ in the unique variances only does not preclude latent variable comparisons. Note that it is best to allow for non-invariances of intercepts or unique variances (as indicated by LMFA) as much as possible in your follow-up analysis, ideally by including states.^39^ Otherwise, the latent means and/or covariances may be estimated incorrectly, especially in the case of large non-invariances (Chen, 2008; Guenole & Brown, 2014). Alternatively, one could perform one analysis per state (using the state-specific factor scores) and weight the observations according to the posterior state-membership probabilities such that observations with larger probabilities receive more weight than observations with lower probabilities. Another option could be to conduct a weighted multilevel analysis, in which the states would be considered as observed groups. Furthermore, if “partial” metric or strong invariance holds (i.e., if only a few loadings or intercepts differ; Byrne et al., 1989), one may exclude non-invariant items or, again, capture the differences by letting parameters differ across states in subsequent analyses or dealing with it by conducting separate analyses with weighted data. Moreover, to avoid non-invariance in future studies, one could consider rephrasing or removing the problematic items from the questionnaire. To conclude, LMFA can be viewed as a primary analysis step that indicates which observations are comparable and what the MMs look like, and that in turn facilitates decisions about how to further analyze the ILD.

## Discussion

When studying dynamics in psychological constructs in intensive longitudinal data (ILD), it is crucial to investigate whether the measurement models (MMs) underlying the responses are invariant across subjects and time, which is easily violated due to between-person differences and/or situation-specific changes in item interpretation and response styles. Undetected measurement non-invariance poses a threat to valid inference from state-of-the-art ILD analyses. In this tutorial, we showed how to explore which MMs underlie the data, what transitions between these MMs look like, and how to investigate whether covariates are related to such transitions with latent Markov factor analysis (LMFA). LMFA identifies which observations are comparable by classifying them into the same MM state, which helps to safeguard valid inferences. Moreover, researchers gain substantive insights into the dynamics of the underlying MM in their ILD.

The package *lmfa* was implemented in the open-source software R to provide researchers with a freely available software option for performing LMFA. Even though this is a huge advantage, it is important to stress that some features are currently not (yet) available. In the following, we will elaborate on the current limitations of the package and ideas for future extensions.^40^ Firstly, the state-specific MMs in step 1 are currently obtained using exploratory factor analysis (EFA). As previously explained, EFA is less restrictive than confirmatory factor analysis (CFA), which implies that it allows the detection of all types of non-invariance in the loadings. However, for some datasets, it is certainly interesting to use a CFA model—thus, with fixed patterns of zero factor loadings. For instance, researchers may want to rely on results from previous research showing that the configural factor structure is relatively stable across subjects and time.

Relatedly, a CFA variant that additionally allows for equality restrictions across the states would enable users to test whether higher levels of (partial) invariance, such as weak invariance, hold across (part of) the states by comparing the BIC (or alternative information criteria) of models with increasingly more restrictions (for a similar procedure for non-dynamic latent classes, see Lubke & Muthén, 2005). However, before including it in the *lmfa* package, the CFA variant of LMFA with equality restrictions still needs to be evaluated in future research.

Secondly, the factor analysis models in step 1 assume continuous item responses. If items are measured with only a few categories or if the item responses are heavily skewed, state-specific “latent trait” (or “item response theory”) models should be employed in step 1 of the analysis to adequately deal with categorical data, as is done in the extension called latent Markov latent trait analysis (LMLTA; Vogelsmeier et al., 2021b). Performing LMLTA is currently only possible in LG, but advanced R users could specify their own state-specific models for step 1 (for instance, by using mixture models for categorical data from other packages) and use the posterior state-membership probabilities as input^41^ for the step 3 analysis with *lmfa*. However, to the best of our knowledge, suitable packages are currently not available in R. If a package becomes available, the possibilities to include it in the *lmfa* package (to perform LMLTA) will be examined.

Thirdly, before performing step 1 in *lmfa*, users have to remove records that contain missing values on some of the indicators, that is, for measurement occasions that were not completely skipped (note that completely omitted measurement occasions are dealt with by the continuous-time approach). As previously stated, removing observations may affect the results and conclusions. Generally, technological advances in many experience sampling methodology apps prevent subjects from submitting incomplete responses. However, sometimes researchers would rather have incomplete data than lose the measurement occasion entirely. Furthermore, missing data may be a result of the increasingly employed “planned missing-data designs”, in which researchers deliberately assess only selected items at each measurement occasion while omitting others to reduce the burden on the subjects, which, in turn, tends to increase the quality of the responses (Silvia et al., 2014; van Roekel et al., 2019). In the future, *lmfa* will be extended to be applicable for ILD collected with such innovative missing-data designs and missing data in general.

Fourthly, the inclusion of covariates in step 3 of the analysis helps researchers understand why certain subjects transition between MMs over time, but some researchers might be more (or also) interested in individual transition patterns, especially in the case of only a few subjects. Estimating subject-specific transition parameters is currently not possible in *lmfa*. However, one can estimate one transition model per subject. More specifically, steps 1 and 2 (i.e., evaluating the MMs and obtaining the state assignments and classification errors) would still be conducted for all subjects^42^, but step 3 would be performed for each subject individually. Additionally, instead of inspecting subject-specific transition parameters, it might be interesting to investigate whether unobserved subgroups of subjects have similar transition patterns, especially in the case of many subjects. Theoretically, it is possible to cluster subjects based on their transition behavior by adding a latent grouping variable to the LMFA in step 3 (e.g., see Crayen et al., 2017; Vogelsmeier et al., 2021b). This is not possible with *lmfa,* but advanced R users may consider using the *depmix* package in step 3 of LMFA by passing the modal state assignments and classification error probabilities as fixed parameters to the depmix() function. This package allows for a latent grouping variable in the transition model but uses a discrete-time latent Markov model and, hence, does not account for differences in intervals, which may impair the estimation of the transition model parameters when intervals are unequal (for details about the syntax and about how to fix parameters, see the package documentation; Visser, 2007).

Lastly, *lmfa* users currently must draw conclusions about (non-)invariance by visually comparing the state-specific MMs. If the number and nature of the factors appear to be the same across states, determining which parameters differ substantially becomes a daunting task, especially when comparing parameters for models with many states and factors. Furthermore, small parameter differences across states will always be found due to sampling fluctuations and error fitting. Deciding which differences are practically or statistically significant is not a trivial problem. On top of that, the states also capture differences in the factor variances (in the loadings) and factor means (in the intercepts) due to the model identification constraints (see “The state-specific measurement models” section). In order to obtain loadings that are optimally comparable across states and to enable hypothesis testing for these loadings (using Wald tests), multigroup factor rotation (MGFR; De Roover & Vermunt, 2019) should be applied. MGFR solves rotational freedom by rotating the loadings towards a simple structure within the states and towards agreement across states while unraveling differences in the loadings from differences in the factor variances. MGFR is currently only available in LG, but the possibility of including it in *lmfa* will be investigated in the future.

Similarly, a solution for optimally comparing intercepts (with hypothesis tests) could be to employ multiple group factor alignment (MGFA; Asparouhov & Muthén, 2014), in which the factors are rescaled and shifted (or “aligned”) with respect to their means, thereby disentangling differences in the intercepts from differences in the factor means. However, currently, MGFA is only applicable to CFA models without cross-loadings. If an MGFA extension for EFA becomes available, possibilities to include the method in *lmfa* will be examined. Until MGFR and MGFA are implemented, users can inspect whether there appears to be a difference in the scaling of all loadings of a factor and/or a “shift” in all intercepts of items that correspond to the same factor (as indicated by loadings that are not close to zero). If separate loadings or intercepts differ across states, it is unlikely that these differences are caused by differences in the underlying factor variances or factor means, respectively.

### Supplementary Information

ESM 1 (DOCX 72 kb)

## Appendix

In the following, we summarize the arguments and the output for each function. Note that additional documentation files are available for all functions. These can be called by typing a questionnaire mark followed by the function name (e.g., ?step1).

### step1() function

**Arguments**

**Table:**

data	The dataset with the indicators.
indicators	The variable names of the indicators.
n_state	The number of states that should be estimated when modelselection = FALSE
n_fact	The number of factors that should be estimated when modelselection = FALSE
modelselection	The indication whether model selection should be performed or not. The default is FALSE.
n_state_range	The range of states that should be estimated when modelselection = TRUE.
n_fact_range	The range of factors that should be estimated when modelselection = TRUE.
n_starts	The number of start sets. Multiple start sets are required in order to increase the chances of finding the global maximum (for details, see Supplementary Material S.3.5). The default is 25.
n_initial_ite	The number of initial iterations, that is, the number of iterations that is performed for each start set (for an explanation, see Supplementary Material S.3.5). The default is 15.
n_m_step	The number of maximization-step iterations inside the implemented expectation maximization algorithm (for details, see Supplementary Material S.3). The default is 10.
em_tolerance	The estimation convergence criterion (for details, see Supplementary Material S.3.4). The default is 1e-8.
m_step_tolerance	The criterion for stopping the maximization-step iterations. The default is 1e-3. Thus, the maximization-step iterations stop when either m_step_tolerance or n_m_step has been reached.
max_iterations	The maximum number of iterations after which the estimation terminates regardless of whether convergence has been reached or not. The default is 1000 iterations.
n_mclust	The number of mclust start sets (for details, see Supplementary Material S.3.5). The default is 5.

**Output**

**Table:**

n_it	The number of iterations.
seconds	The time in seconds that was required to reach convergence.
convergence	Indicates whether the model estimation converged prior to reaching the maximum number of iterations. A convergence of 1 indicates that the model converged.
LL	The value of the loglikelihood (for details, see Supplementary Material S.3.1).
BIC	The value of the BIC (for details, see Supplementary Material S.6.2).
intercepts	The state-specific intercepts.
loadings_stand_obli	The state-specific standardized obliquely rotated loadings. If the number of factors is equal to one, the loadings are equal to the ones in loadings_stand_list.
unique_variances	The state-specific unique variances.
state_proportions	The state proportions.
n_obs	The total number of observations across all subjects and time-points.
n_par	The total number of free parameters (for details, see Supplementary Material S.6.1).
explained_variance	The amount of explained variance weighted by the state sizes (for details, see Supplementary Material S.6.4).
n_state	The number of states.
n_fact	The state-specific number of factors.
intercepts_list	List of state-specific intercepts.
loadings_list	List of state-specific loadings.
loadings_stand_list	List of state-specific standardized loadings.
loadings_obli_list	List of state-specific obliquely rotated loadings. If the number of factors is equal to one, the loadings are equal to the ones in loadings_list.
loadings_stand_obli_list	List of state-specific standardized obliquely rotated loadings. If the number of factors is equal to one, the loadings are equal to the ones in loadings_stand_list.
unique_variances_list	List of state-specific unique variances.
factor_correlations_stand_obli_list	List of state-specific factor correlations resulting from the rotations of the standardized loadings.
factor_correlations_obli_list	List of state-specific factor correlations resulting from the rotations of the loadings.
activated_contraints	The number of activated constraints (for details, see Supplementary Material S.3.4).
classification_posteriors	The posterior state-membership probabilities and the modal state assignments.
classification_errors	The classification errors when using the modal state assignment (for details, see Supplementary Material S.4).
classification_errors_prob	The classification-error probabilities when using the modal state assignment (for details, see Supplementary Material S.4).
R2_entropy	The entropy-based R-squared measure (for details, see Supplementary Material S.6.6)
warning_loadings	A message indicating whether convergence for rotating the loadings was reached or not.
warning_loadings_stand	A message indicating whether convergence for rotating the standardized loadings was reached or not.
raw_data	The data corresponding to the indicator items that were used in the analysis.

### step2() function

**Arguments**

**Table:**

data	The dataset used in step1().
model	The model estimated with step1().

**Output**

**Table:**

classification_posteriors	The posterior state-membership probabilities and the modal state assignments.
classification_errors	The classification errors when using the modal state assignment (for details, see Supplementary Material S.4).
classification_errors_prob	The classification-error probabilities when using the modal state assignment (for details, see Supplementary Material S.4).
R2_entropy	The entropy-based R-squared measure (for details, see Supplementary Material S.6.6).
totoal_classification_error	The total classification error.
state_proportions	The state proportions.
data	The data with the posterior state-membership probabilities and the modal state assignments attached.

### step3() function

**Arguments**

**Table:**

data	The dataset (including the covariate values).
timeintervals	The name of the column containing the intervals between measurement occasions. The default is NULL, which means that the measurement occasions are assumed to be equidistant.
identifier	The name of the column containing the subject identifiers.
n_state	The number of states that was used for the estimation with step1().
postprobs	The posterior state-membership probabilities of step2().
transitionCovariates	The covariate(s) for the transition intensities. The default is NULL, which means that no covariate effects are estimated.
initialCovariates	The covariate(s) for the initial state probabilities. The default is NULL, which means that no covariate effects are estimated.
n_starts	The number of random start sets (for details, see Supplementary Material S.5.4). The default is 25.
n_initial_ite	The number of initial iterations that should be performed for each start set. The default is 10.
method	The estimation method. The default is "BFGS", which is usually faster and more stable when including covariates. The alternative is "CG".
max_iterations	The maximum number of iterations after which the estimation stops regardless of whether convergence has been reached or not. The default is 1000.
tolerance	The convergence tolerance (for details, see Supplementary Material S.5.3). The default is 1e-10. When the message occurs that the model has likely not converged because the Hessian is not positive definite, it is advisable to set the argument to a lower value and repeat the analysis (e.g., 1e-16; Jackson, 2011).
scaling	A scaling parameter for the loglikelihood that can prevent numerical problems from occurring, which is internally passed to the optimization function optim(). An appropriate scale value is close to -2 times the loglikelihood, but the loglikelihood is of course unknown prior to estimating the model. Therefore, by default, *lmfa* uses an approximation, which is based on the loglikelihood value of a CT-LMM without transitions. Next to this default (i.e., scaling = "proxi"), it is also possible to specify own scale values.

**Output**

**Table:**

seconds	The time in seconds that was required to reach convergence.
convergence	Indicates whether the model estimation converged prior to reaching the maximum number of iterations. A convergence of 1 indicates that the model converged. Note that it is not possible to obtain the number of iterations because this information is not returned by the optim() function.
LL	The value of the loglikelihood (for details, see Supplementary Material S.5.1).
BIC	The value of the BIC (for details, see Supplementary Material S.6.9).
WaldTests	The Wald test output.
estimates	The parameter estimates of the transition model.
classification_posteriors	The posterior state-membership probabilities and the modal state assignments.
state_proportions	The state proportions.
n_initialCovariates	The number of covariates for the initial state probabilities.
n_transitionCovariates	The number of covariates for the transition intensities.
n_initial_covariates	The number of covariates specified for the initial state parameters.
transition_covariate_means	The number of covariates specified for the transition parameters.
n_obs	The total number of observations across all subjects and time-points.
n_par	The total number of free parameters (for details, see Supplementary Material S.6.8).
n_state	The number of states.
data	The data with the posterior state-membership probabilities and the modal state assignments attached.

### chull_lmfa() function

**Arguments**

**Table:**

x	The model-selection output of the function step1().

**Output**

Prints the models on the upper boundary of the CHull, the corresponding scree-test values, and the selected model(s).

### factorscores_lmfa() function

**Arguments**

**Table:**

data	The dataset used in step1().
model	The model estimated with step1().
oblique	The indication whether the factor scores should be obtained for the obliquely rotated loadings or unrotated loadings. The default is TRUE, indicating that the obliquely rotated loadings are considered.
rounding	The number of decimals to which the results should be rounded. The default is 4.

**Output**

Attached the state-specific factor scores to the dataset.

### probabilities() function

**Arguments**

**Table:**

model	The transition-model output of the function step3().
deltaT	The interval for which the transition probabilities should be calculated.
initialCovariateScores	The covariate scores for which the probabilities should be calculated. The default is NULL, which implies that any scores are set equal to the sample means.
transitionCovariateScores	The covariate scores for which the probabilities should be calculated. The default is NULL, which implies that any scores are set equal to the sample means.
rounding	The number of decimals to which the results should be rounded. The default is 2.

**Output**

Prints the initial state and transition probabilities for specified covariate values (and intervals).

### invariance() function

**Arguments**

**Table:**

model	The transition-model output of the function step3().
identifier	The name of the column containing the subject identifiers.

**Output**

Prints, for each state, the identification number of subjects who are in that state during the entire participation period.

## Footnotes

1 Note that one may also investigate ordinal data as long as the data has more than a few response categories (say at least five; Rhemtulla et al., 2012) and the distribution across the categories is approximately normal. However, the influence on the parameter estimation has not yet been investigated for LMFA in particular, and results should therefore be interpreted with caution. Highly skewed responses with only a few response categories may lead to convergence problems and local optima (for a description of local optima, see (“Increasing the chance to find the global maximum and assessing convergence” section). For such data, the LMFA extension to categorical data should be used, that is, latent Markov latent trait analysis (Vogelsmeier et al., 2021b).

2 The intervention was either personalized lifestyle advice or the advice in combination with a skydive. For simplicity in this tutorial, we do not distinguish between different types of interventions.

3 Note that both estimation approaches are available in LG.

4 Note that *lmfa* users do not have to calculate any probabilities themselves, as the package provides them.

5 For an estimate of the required sample size, users may consult guidelines for multinomial logistic regression (e.g., de Jong et al., 2019).

6 Note that the probabilities of transitioning away from a state increase for increasing interval lengths only up to a certain point, where the probabilities asymptote (for details including a graphical illustration, see Vogelsmeier et al., 2019a).

7 For readers familiar with survival models, note that the intensities are actually equivalent to hazard rates (Cox & Miller, 1965; Kalbfleisch & Lawless, 1985; Kleinbaum & Klein, 2012).

8 More specifically, the probabilities are equal to the matrix exponential of the product of the intensities and the interval (see Supplementary Material S.2.1).

9 Note that, for time-varying covariates, the score at time-point *t* is used to predict the transition probabilities from time-point *t* − 1 to time-point *t*. This makes most sense for LMFA because MMs are typically triggered by momentary circumstances (e.g., social interactions). Even when assuming temporal precedence (e.g., the effect of perceiving a negative event prior to time-point *t*), questionnaires are usually designed in a way that they ask subjects about such covariates at the current time point (e.g., “Please rate the unpleasantness of the most unpleasant event you have experienced since the previous measurement occasion”).

10 Note that it is theoretically also possible to use a “proportional assignment”, which assigns the state memberships according to the posterior state-membership probabilities. However, the proportional assignment is unfeasible for data that contain a large number of measurement occasions for many subjects (Di Mari et al., 2016) and, thus, for ILD.

11 Note that the package consists of only six functions in total (next to the general summary() and plot() functions). An overview of these functions is provided in the Appendix.

12 The reason is that the value of the transition intensities (for covariate scores being equal to zero) are directly related to the size of the unit (note that the model fit is not influenced by the unit, however). More specifically, the larger the size of the unit, the smaller the intensities and the more likely numerical problems occur.

13 Future extensions of *lmfa* to deal with missing data inside the estimation are discussed in “Discussion” section.

14 It is important to note that the model selection takes a considerable amount of time. For instance, the time required to estimate all the models for the example data (i.e., 14 model; also see “Model selection with *lmfa*” section) took around three hours on a desktop PC with an Intel Xeon Gold 6130 CPU 2.10 GHz, 32 GB memory).

15 Note that the function contains additional arguments related to the estimation procedure for which default values are provided. These values may be changed by the user if desired. For an explanation of the additional arguments, see “step1() function” section and the function documentation, which can be called with ?step1.

16 Note that the number of parameters is equal to the sum of the state-specific intercepts, unique variances, and loadings and the state proportions minus 1 (minus 1 because one state is treated as a reference state; see Supplementary Material S.6.1).

17 For details about the function, see “chull_lmfa() function” section or call the documentation file with ?chull_lmfa.

18 The second-best model according to the scree value is model [3 3 2 2]. However, visual inspection of the CHull shows that adding a fourth state does not improve the fit considerably. The next-best model is model [3 2]. If we inspected model [3 2], we would see that the smallest state (i.e., the neutral state) would be divided into the pleasure and the displeasure state.

19 Note that, in the summary() function, the user can specify an additional argument to change the number of decimals to which the parameters should be rounded. The default for the summary of the MM parameters is  .

20 Note that the model object (i.e., measurementmodel323) contains additional information that is not directly relevant for the interpretation, but that may be interesting for some users (e.g., unrotated and unstandardized loadings). For an overview of all available output, see “step1() function” Section.

21 Note that, for clarity, loadings with absolute values larger than .3 are printed in boldface in this tutorial. However, the normal output in R does not include any boldface loadings.

22 More specifically, they are standardized by means of the standard deviations of the item scores across all states.

23 If desired, however, the user can also request unstandardized and unrotated loadings.

24 Note that rules of thumb are only rough guidelines, and making decisions about considerable loadings with the aid of standard errors is considered more justifiable (Zhang, 2014). However, standard errors are not yet available in *lmfa*.

25 In *lmfa*, the factor scores are calculated by means of the regression method (Thomson, 1934; Thurstone, 1935), which is one of the most commonly used approaches. For the exact computation, see Supplementary Material S.6.9. Note that the calculation of factor score estimates is generally considered controversial because different methods can result in (very) different scores (which is referred to as the problem of factor score indeterminacy; for discussions on this and possible solutions to account for biases in subsequent analyses, see, e.g., Devlieger et al., 2016; Green, 1976; Grice, 2001).

26 In the factorscores_lmfa() function, the user can specify two additional arguments. The first indicates whether the factor score estimates should be obtained for the obliquely rotated loadings. The default is  . Otherwise, the factor score estimates corresponding to the unrotated factor loadings are obtained. The second argument pertains to the number of decimals to which the factor score estimates should be rounded. The default is  . For details about the function, see “factorscores_lmfa() function” section or call the documentation file with ?factorscores_lmfa.

27 For an additional explanation of the function arguments, see “step2() function” section or call the documentation file with ?step2.

28 Again, the user can adjust the number of decimals to which the parameters should be rounded. The default for the summary of the classification outputs is  .

29 The reason is that the ${R}_{entropy}^2$ tends to be overestimated for bad state separations (e.g., if we find a value of .4, the real state separation is probably even lower). In turn, the classification errors are underestimated, leading to an incorrect correction in the final step of the analysis (Vermunt, 2010).

30 Note that the results of the step3() function are sensitive to start values for the transition intensities (see Supplementary Material S.5.4). Therefore, one should use at least 25 start sets.

31 If the maximum number of iterations is reached without convergence, *lmfa* displays a note with advice to repeat the estimation with an increased number of max_iterations.

32 Note that the user may change the defaults of additional arguments pertaining to the estimation procedure. For an explanation of these arguments, see “step3() function” section and the function documentation that can be called with ?step3.

33 Again, the user can change the number of decimals to which the parameters should be rounded. The default for the summary of the transition model is  . Thus, by default, there are two more decimals than for the other representations. This is because some parameters can become very small such that differences would vanish too quickly when using fewer decimal points.

34 Note that the number of degrees of freedom equals the number of regression coefficients when including the covariate (e.g., *df* = 6 when testing the overall significance of a covariate with effects on six transition intensities; see Supplementary Material S.6.7.)

35 An additional explanation of the arguments can be found in “probabilities() function” section and in the function documentation, which can be called with ?probabilities.

36 The user can determine an additional argument in the probabilities() function to change the number of decimals to which the parameters should be rounded. The default for the probabilities is  .

37 All results were in line with the data-generating model.

38 When proceeding with factor scores from one state, it is important to investigate the certainty of the final state assignments. For instance, the largest posterior state probabilities for some observations might be rather low (say, below .6 or .7), indicating that it is less clear which of the state-specific MMs best fits the scores of the observation. Therefore, it is advisable to remove these observations before conducting further analyses.

39 Note that this is possible with more advanced analyses like dynamic latent class analysis (DLCA; Asparouhov et al., 2016). Moreover, in some analyses, it is at least possible to allow for MM differences across subjects, for example in dynamic structural equation modeling (McNeish & Hamaker, 2020; McNeish et al., 2021).

40 Note that the commercial software LG offers most of the features discussed below.

41 As described in “Covariate selection procedure using Wald tests” section, this would be done by providing a data frame with posterior state-membership probabilities per state via the argument postprobs.

42 One may also perform the entire LMFA for data of a single subject if the number of observations is large enough. For guidelines about the required number of observations, see Vogelsmeier et al. (2019b).
