# Supplementary material for: How to explore within-person and between-person measurement model differences in intensive longitudinal data with the R package lmfa
Source: Behav Res Methods. 2022 Sep 1;55(5):2387–422. doi: 10.3758/s13428-022-01898-1 (PMC10439104; doi:10.3758/s13428-022-01898-1)
Supplement: Supplementary file 1 — (DOCX 72 kb) [file 13428_2022_1898_MOESM1_ESM.docx]

Supplementary Material for the Manuscript:

**How to explore within-person and between-person measurement model differences in intensive longitudinal data with the R package *lmfa***

Vogelsmeier, Leonie V.D.E, Vermunt, Jeroen K., De Roover, Kim

Tilburg University, The Netherlands

# Online Supplement

This online supplement provides all technical information about latent Markov factor analysis (LMFA) and the three-step (3S) estimation with the R package *lmfa*. We first introduce relevant data notation (S.1) in the following. Then, we explain LMFA (S.2). After that, we describe the three estimation steps (as presented in the main body of the paper) and how they are implemented in *lmfa* (S.3–S.5). Finally, we provide equations for relevant statistics, such as the Bayesian information criterion (BIC; S.6).

## Data Notation

The observations are denoted by $y_{ijt}$ with $i=1,\ldots,I$ referring to the subjects, with $j=1,\ldots,J$ indicating the variables, and with $t=1,\ldots,T$,^[[1]](#footnote-1)^ referring to the measurement occasions. The observations are collected in the $J\times1$ vectors $\mathbf{y}_{it}=\left( y_{i1t}, y_{i2t},\ldots, y_{iJt} \right)'$ that are themselves stored in the $T\times J$ data matrices $\mathbf{Y}_{i}=\left( \mathbf{y}_{i1}^{'}, \mathbf{y}_{i2}^{'},\ldots, \mathbf{y}_{iT}^{'} \right)'$. The covariate scores are denoted by $z_{itu}$ (with $u=1,\ldots,U$ referring to the covariates) and are collected in the $U\times1$ vectors $\mathbf{z}_{it}=\left( z_{it1}, z_{it2},\ldots, z_{itU} \right)'$, which are themselves collected in the $T\times J$ matrices $\mathbf{Z}_{i}=\left( \mathbf{z}_{i1}^{'}, \mathbf{z}_{i2}^{'},\ldots, \mathbf{z}_{iT}^{'} \right)$.

## LMFA

The LMFA model consists of a transition model (i.e., the continuous-time (CT-)LMM) and state-specific measurement models (MMs) (i.e., the FA models). The conceptual ideas behind the models were described in the main body of the paper. In the following, we explain the technical details.

### CT-LMM

The CT-LMM makes two assumptions. First, the first-order Markov assumption states that the probability of being in state $k$ (with $k=1,\ldots,K$) at time-point $t$ depends only on the state-membership $l$ (with $l=1,\ldots,K$) at time-point $t-1$. Second, the local independence assumption states that the responses $\mathbf{y}_{it}$ at time-point $t$ depend only on the state-membership $k$ at this time-point. The CT-LMM, for subject $i$, is defined as follows:

|  | $p\left( \mathbf{Y}_{i},\mathbf{S}_{i}\mathbf{\vert}\mathbf{Z}_{i} \right)=p\left( \mathbf{y}_{i1},\ldots,\mathbf{y}_{iT,}\mathbf{s}_{i1}, \ldots, \mathbf{s}_{iT}\vert\mathbf{z}_{i1},\ldots,\mathbf{z}_{iT} \right)=\overset{\begin{aligned} initial state \\ probabilities \end{aligned}}{\overbrace{p\left( \mathbf{s}_{i1} \vert\mathbf{z}_{i1} \right)}}\prod_{t=2}^{T} \overset{\begin{aligned} transition \\ probabilities \end{aligned}}{\overbrace{p_{\delta_{ti}}\left( \mathbf{s}_{it} \vert{\mathbf{s}_{it-1}\mathbf{,z}}_{it} \right)}}\prod_{t=1}^{T} \overset{\begin{aligned} response \\ probabilities \end{aligned}}{\overbrace{p\left( \mathbf{y}_{it} \vert\mathbf{s}_{it} \right)}}.$ | (S1) |
| --- | --- | --- |

The $K\times1$ vectors $\mathbf{s}_{it}=\left( s_{it1},\ldots,s_{itK} \right)^{'}$ contain binary indicators that determine the state memberships at time-point $t$, with $s_{itk}=1$ for one state $k$ and $s_{itk}=0$ for all other states. As can be seen in Equation S1, the model contains three types of probabilities:

1. $p\left( \mathbf{s}_{i1} | \mathbf{z}_{i1} \right)$ is a $K\times1$ vector with the initial state probabilities that define the probabilities to start in a certain state at the first time-point and thus sum to one.
2. $p_{\delta_{ti}}\left( \mathbf{s}_{it} | {\mathbf{s}_{it-1}\mathbf{,z}}_{it} \right)$ is a $K\times K$ matrix $\mathbf{P}_{\delta_{ti}}$with transition probabilities that define the probabilities of staying in a state or transitioning to another state at two consecutive measurement occasions with row sums equal to 1. As indicated by the index $\delta_{ti}$, the transition probabilities are a function of the interval $\delta_{ti}$ between two consecutive observations and the $K\times K$ transition intensity matrix $\mathbf{Q}$ with the off-diagonal elements $q_{lk}=\lim_{\delta\to0} \frac{p(s_{itk}=1|s_{it-\delta,l}=1, \mathbf{z}_{it})}{\delta}$, which define transitions between the origin state $l$ and the destination state $k$ for a very small time unit. The diagonal elements equal the negative row sums (Cox & Miller, 1965). The transition probability matrix $\mathbf{P}_{\delta_{ti}}$ is obtained with $\mathbf{Exp}\left( \boldsymbol{Q\times}\delta_{ti} \right)$, where $\mathbf{Exp}\left( \boldsymbol{\cdot} \right)$ denotes the matrix exponential.
3. $p\left( \mathbf{y}_{it} | \mathbf{s}_{it} \right)$ determines the state-specific response probabilities and, thus, the probabilities of having a certain response pattern depending on the state membership at time-point $t$. In LMFA, the probabilities depend on the state-specific MMs.

As can be seen from Equation (S1), the initial state and transition intensities may depend on the covariates $\mathbf{z}_{it}$. Note that the covariates and the observations at time-point $t$ are assumed to be conditionally independent given the state-membership at that time-point (i.e., the covariates affect only the state-membership and not the indicators directly). The covariates are included by means of regression (Bartolucci, Farcomeni, & Pennoni, 2014; Vermunt, Langeheine, & Böckenholt, 1999). Note that a logit model is used for the initial state probabilities, and a log-linear model is used for the transition intensities. Specifically, the initial state probabilities are modeled as:

|  | $\log\frac{p(s_{i1k}=1\vert\mathbf{z}_{i1})}{{p(s}_{i11}=1\vert\mathbf{z}_{i1})}=\beta_{0k}+\boldsymbol{\beta}_{k}^{'}\mathbf{z}_{it=1}; \mathrm{for}k=2,\ldots,K,$ | (S2) |
| --- | --- | --- |

with $\beta_{0k}$ indicating the initial state intercepts and $\boldsymbol{\beta}_{k}^{'}=\left( \beta_{k,z_{i11}},\ldots,\beta_{k,z_{i1U}} \right)'$ indicating the slopes (i.e., the covariate effects). For the transition intensities, the model is

|  | $\log q_{lk}=\gamma_{0lk}+\boldsymbol{\gamma}_{lk}^{'}\mathbf{z}_{it};$ $\mathrm{for}k\neq l,$ | (S3) |
| --- | --- | --- |

with $\gamma_{0lk}$ as transition intercepts and $\boldsymbol{\gamma}_{lk}^{'}=\left( \gamma_{lk,z_{it1}},\ldots,\gamma_{lk,z_{itU}} \right)$ as slopes.

### State-specific FA models

The state-specific FA models determine what the MMs look like. The state-specific FA model for subject $i$ being in state $k$ at time-point $t$ (i.e., $s_{itk}=1$) is:

|  | $[\mathbf{y}_{it}\vert s_{itk}=1]= \boldsymbol{\nu}_{k}+\boldsymbol{\Lambda}_{k} \mathbf{f}_{it}+ \mathbf{e}_{it}$, | (S4) |
| --- | --- | --- |

where $\boldsymbol{\nu}_{k}$ is a state-specific $J \times1$ intercept vector; $\boldsymbol{\Lambda}_{k}$ is a state-specific $J \times F_{k}$ loading matrix, where $F_{k}$ indicates the number of factors for state *k*;$\mathbf{f}_{it}\boldsymbol{\sim}MVN(\boldsymbol{0},\boldsymbol{\Phi}_{k})$ is a subject-specific and time-point-specific $F_{k}\times1$ vector of factor scores with $\boldsymbol{\Phi}_{k}$ being the state-specific factor covariance matrix. Furthermore, $\mathbf{e}_{it} \sim MVN(\boldsymbol{0},\boldsymbol{\Psi}_{k})$ is a subject- and time-point-specific $J \times1$ vector of residuals, where $\boldsymbol{\Psi}_{k}$ contains the unique variances $\psi_{kj}$ on the diagonal and zeros on the off-diagonal*.* This implies that the response probabilities, $p\left( \mathbf{y}_{it} | \mathbf{s}_{it} \right)$ in Equation S1 are determined by state-specific multivariate normal distributions with covariance matrices $\boldsymbol{\Sigma}_{k}=\boldsymbol{\Lambda}_{k}\boldsymbol{\Phi}_{k}\boldsymbol{\Lambda}_{k}^{'}+\boldsymbol{\Psi}_{k}$ and mean vectors $\boldsymbol{\nu}_{k}$.

## Step 1 in *lmfa*

The first step of the 3S procedure consists of obtaining the maximum likelihood (ML) estimates for the state-specific MMs (and thus the FA models) using mixture factor analysis (McLachlan & Peel, 2000; McNicholas, 2016). In *lmfa*, this is done through the step1() function. Details about the arguments and the output are provided in the Appendix of the paper. In the following, we first show the loglikelihood ($\log L$) function that must be optimized. Then, we explain how the model is estimated in the *lmfa* package, followed by the description of the inherent convergence criteria, the implemented algorithm, and, finally, the multistart procedure.

### Likelihood function

In order to obtain the ML estimates, the following $\log L$ function has to be maximized:

|  | ${\log L}_{STEP1}=\sum_{i=1}^{I} \sum_{t=1}^{T} \log\left( \sum_{k=1}^{K} p\left( s_{itk}=1 \right)p\left( \mathbf{y}_{it}\vert s_{itk}=1 \right) \right),$ | (S5) |
| --- | --- | --- |

where $p\left( s_{itk}=1 \right)$ are the state proportions, $p\left( \mathbf{y}_{it}|s_{itk}=1 \right)=MVN\left( \mathbf{y}_{it}\boldsymbol{|}\boldsymbol{\nu}_{k}\boldsymbol{,}\boldsymbol{\Sigma}_{k} \right)$ are the response probabilities for a specific state, and $\boldsymbol{\Sigma}_{k}$ was defined before (S.2.2). In step1(), the proportions and response probabilities are estimated using an expectation-maximization (EM) algorithm described next.

### Estimation

The observed-data $\log L$ in Equation (S5) is complicated by the latent-state memberships and the latent factor scores. The EM algorithm solves this problem by iterating through the following steps. First, in the expectation- (E-)step, the model parameters are assumed to be given, and the posterior state-membership probabilities are calculated accordingly (i.e., under current estimates of the model parameters). Subsequently, in the maximization- (M-)step, the posterior state-membership probabilities are treated as observed, and the model parameters are updated (i.e., optimized) one by one. In fact, in the M-step, another EM algorithm with a limited number of iterations is used to update the factor parameters for each state. The algorithm iterates through the E- and M-steps until convergence. In the following, we describe the specific steps of the EM algorithm of *lmfa* (S.3.4), including convergence criteria (S.3.3) and the multistart procedure (S.3.5).

### Convergence

The EM algorithm (S.3.4) stops when reaching a convergence criterion. In the step1() function, the convergence is evaluated with respect to both the ${\log L}_{STEP1}$:

|  | $\Delta_{logL}=$ ${\log L}_{STEP1}^{v}-{\log L}_{STEP1}^{v-1}$, | (S6) |
| --- | --- | --- |

where $v$ refers to the iteration number, and with respect to the sum of the absolute changes in the parameter estimates:

|  | $\Delta_{\hat{\theta}_{EM}}=\sum_{r=1}^{R} \left\vert\frac{\hat{\theta}_{r}^{v}-\hat{\theta}_{r}^{v-1}}{\hat{\theta}_{r}^{v-1}} \right\vert,$ | (S7) |
| --- | --- | --- |

with $r=1,\ldots,R$ referring to the separate parameters. More specifically, the algorithm stops when one of the two criteria (or the maximum number of specified iterations) is reached. Within the EM algorithm, the M-step is either repeated as long as the parameters still change a lot, which is evaluated with

|  | $\Delta_{\hat{\theta}_{M-step}}=\sum_{r=1}^{R} \left\vert\frac{\hat{\theta}_{r}^{b}-\hat{\theta}_{r}^{b-1}}{\hat{\theta}_{r}^{b-1}} \right\vert,$ | (S8) |
| --- | --- | --- |

where $b$ refers to the M-step iteration number, or until a certain number of M-step iterations is reached.

### EM algorithm

In the following description of the EM algorithm, we use specific values to specify, for instance, the convergence tolerance and the maximum number of iterations. These are just the default values, which the user may change.

**START**

Set the iteration number: $v=0$ (or equal to the final iteration number of the chosen start set as described in S.3.5). **While** $\Delta_{logL}$ and $\Delta_{\hat{\theta}_{EM}}$ $>1e-06$ and $v<1000$:

1. Update the iteration number: $v=v+1$.
2. Update posterior state-membership probabilities $p\left( s_{itk}=1|\mathbf{y}_{it} \right)$ as follows:

|  | $p\left( s_{itk}=1\vert\mathbf{y}_{it} \right)=\frac{p\left( s_{itk}=1 \right)p\left( \mathbf{y}_{it}\vert s_{itk}=1 \right)}{p\left( \mathbf{y}_{it} \right)}=\frac{p\left( s_{itk}=1 \right)p\left( \mathbf{y}_{itk}\vert s_{itk}=1 \right)}{\sum_{k=1}^{K} p\left( s_{itk}=1 \right)p\left( \mathbf{y}_{itk}\vert s_{itk}=1 \right)}.$ | (S9) |
| --- | --- | --- |

1. Update the state-specific expected sample size $N_{k}$, state proportions $p\left( s_{itk}=1 \right)$, and $J\times1$ state-specific intercept vectors $\boldsymbol{\nu}_{k}$ with

|  | $N_{k}=\sum_{i=1}^{I} \sum_{t=1}^{T} p\left( s_{itk}=1\vert\mathbf{y}_{it} \right),$ | (S10) |
| --- | --- | --- |
|  | $p\left( s_{itk}=1 \right)=\frac{N_{k}}{I\times T}, \mathrm{and}$ | (S11) |
|  | $\boldsymbol{\nu}_{k}=\frac{\sum_{i=1}^{I} \sum_{t=1}^{T} \left[ p\left( s_{itk}=1\vert\mathbf{y}_{it} \right)\mathbf{y}_{it} \right]}{N_{k}}.$ | (S12) |

1. Compute the $J\times J$ state-specific expected observed covariance matrices $\mathbf{C}_{k}$ with

|  | $\mathbf{C}_{k}=\frac{\sum_{i=1}^{I} \sum_{t=1}^{T} \left[ p\left( s_{itk}=1\vert\mathbf{y}_{it} \right)\left( \mathbf{y}_{it}\boldsymbol{-}\boldsymbol{\nu}_{k} \right)\left( \mathbf{y}_{it}\boldsymbol{-}\boldsymbol{\nu}_{k} \right)\boldsymbol{'} \right]}{N_{k}}.$ | (S13) |
| --- | --- | --- |

1. Set the M-step iteration number: $b=0$.
2. While $\Delta_{\hat{\theta}_{M-step}}>1e-03$ and $b<10$:
   1. Update the M-step iteration number: $b=b+1$.
   2. Compute the $F_{k}\times J$ regression-weight vectors $\boldsymbol{\beta}_{k}$ (from regressions of the latent factors on the items) and the $F_{k}\times F_{k}$ matrices $\boldsymbol{\Theta}_{k}$ containing the expectations of the factor covariances based on the current parameters and the observed data with

|  | $\boldsymbol{\beta}_{k}={\boldsymbol{\Lambda}_{k}^{'}\left( \boldsymbol{\Lambda}_{k}\boldsymbol{\Lambda}_{k}^{'}+\mathbf{D}_{k} \right)}^{-1}$ and | (S14) |
| --- | --- | --- |
|  | $\boldsymbol{\Theta}_{k}\boldsymbol{=}\mathbf{I}_{F_{k}}-\boldsymbol{\beta}_{k}\boldsymbol{\Lambda}_{k}+\boldsymbol{\beta}_{k}\mathbf{C}_{k}\boldsymbol{\beta}_{k}^{'}.$ | (S15) |

- 1. Update loadings $\boldsymbol{\Lambda}_{k}$ and unique variances $\boldsymbol{\Psi}_{k}$ with

|  | $\boldsymbol{\Lambda}_{k}={\mathbf{C}_{k}\boldsymbol{\beta}_{k}^{'}\boldsymbol{\Theta}}_{k}^{-1} \mathrm{and}$ | (S16) |
| --- | --- | --- |
|  | $\boldsymbol{\Psi}_{k}\boldsymbol{=}diag\left( \mathbf{C}_{k}\boldsymbol{-}\boldsymbol{\Lambda}_{k}\boldsymbol{\beta}_{k}\mathbf{C}_{k} \right).$ | (S17) |

Updating the loadings and unique variances comes down to calculating the regression parameters and residual variance in a series of simple linear regressions for items with the factors as predictors. Note that small unique variances can lead to numerical problems. Therefore, if the variances fall below a certain threshold value, they are fixed to this value. The item-specific threshold values are constant across states and determined by multiplying the observed variance of an item with 1e-06.^[[2]](#footnote-2)^

- 1. Compute the change in parameters $\Delta_{\hat{\theta}_{M-step}}$ (Equation (S8)).

1. Compute the ${\log L}_{STEP1}$ value (Equation (S5)).
2. Compute the change in parameters $\Delta_{\hat{\theta}_{EM}}$ and ${\log L}_{STEP1}$ $\Delta_{logL}$ (Equations (S7) and (S6)).

**END**

### Multistart procedure

In order to increase the chances of finding the global maximum, the following multistart procedure with multiple start sets is used, where the number of starts is equal to the specified number of model-based clusterings plus ten times the specified number of start sets (i.e., by default, $5+10\times25=255$). Specifically, the procedure starts with five model-based clusterings by means of the *mclust* package (Scrucca, Fop, Murphy, & Raftery, 2016) to assign observations to states. Then, parameters are initialized for each of the five start sets as follows. The state-specific sample size $N_{k}$, state proportions $p\left( s_{itk}=1 \right)$, and state-specific intercepts $\boldsymbol{\nu}_{k}$, as well as the state-specific weighted sample covariance matrices $\mathbf{C}_{k}$ are computed as in Equations (S12) and (S13). Subsequently, probabilistic principal component analysis (Tipping & Bishop, 1999) is used in order to obtain the state-specific loading matrices $\boldsymbol{\Lambda}_{k}$ and unique variances $\boldsymbol{\Psi}_{k}$. To this end, the following eigendecomposition is conducted:

|  | $\tilde{\mathbf{C}_{k}}=\mathbf{M}_{k}\mathbf{v}_{k}{\mathbf{M}_{k}}^{-1},$ | (S18) |
| --- | --- | --- |

where the state-specific $J\times J$ matrix $\mathbf{M}_{k}$ is the matrix of eigenvectors and the state-specific $J\times1$ vector $\mathbf{v}_{k}$ the eigenvalues. Moreover, the state-specific $J\times F_{k}$ matrix $\mathbf{M}_{k,F_{k}}$contains the first $F_{k}$ eigenvectors, the state-specific $J\times{(J-F}_{k}$) matrix $\mathbf{M}_{k,{-F}_{k}}$contains the disregarded eigenvectors, and the state-specific $F_{k}\times F_{k}$ diagonal matrix $\mathbf{V}_{k,F_{k}}$ contains the first $F_{k}$ eigenvalues of $\mathbf{v}_{k}$ on its diagonal. The loadings and unique variances are then obtained with

|  | $\boldsymbol{\Lambda}_{k}=\mathbf{M}_{k,F_{k}}\sqrt{\mathbf{V}_{k,F_{k}}\boldsymbol{-}\frac{\sum\mathbf{M}_{k,{-F}_{k}}}{J-F_{k}}\mathbf{I}_{F_{k}}}$ and with | (S19) |
| --- | --- | --- |
|  | $\boldsymbol{\Psi}_{k}=\frac{\sum\mathbf{M}_{k,{-F}_{k}}}{J-F_{k}}\mathbf{I}_{J}\boldsymbol{,}$ | (S20) |

where $\sum\mathbf{M}_{k,{-F}_{k}}$ denotes the sum of the disregarded eigenvalues and $\mathbf{I}_{F_{k}}$ and $\mathbf{I}_{J}$ denote $F_{k}\times F_{k}$ and $J\times J$ identity matrices, respectively. Next, the loadings $\boldsymbol{\Lambda}_{k}$ and unique variances $\boldsymbol{\Psi}_{k}$ are updated once as in Equations (S16) and (S17). Subsequently, the value of ${\log L}_{STEP1}$ is obtained (Equation (S5)). Then, the partitions are ranked according to their ${\log L}_{STEP1}$ values.

From the *mclust* start set with the largest ${\log L}_{STEP1}$ value, 250 random start sets are generated by iteratively reassigning 30 percent of the assignments; that is, for start set 1, 30 percent of the *mclust* assignments are reassigned, for start set 2, 30 percent of the assignments from start set 1 are reassigned, and so on. Then, for each of the start sets, parameters are again initialized and the ${\log L}_{STEP1}$ values are computed as described above. Next, the partitions of the random start sets and the best *mclust* set are ranked according to their ${\log L}_{STEP1}$ values and the best 25 start sets (i.e., the number of specified start sets) are selected as start partitions. For each start set, 15 iterations are performed by the EM algorithm (S.3.4). Subsequently, the testing strategy selects the start set with the highest ${\log L}_{STEP1}$ and saves the parameter estimates, $\hat{\theta}_{r}^{best}$, which serve as the initial values in the EM algorithm. Note that, instead of setting the number of iterations $v=0$ (as at the beginning of the first iterations through the start sets), the algorithm continues with the number of iterations that have already been performed; that is $v=15$.

## Step 2 in *lmfa*

In step 2, the subject- and time-point-specific observations are classified into the states $\mathbf{w}_{it}=\left( w_{it1},\ldots,w_{itK} \right)$ based on the largest estimated posterior probability to belong to a state (i.e., based on a so-called “modal” state assignment). Thus, the observations are assigned to the MM that is most likely underlying the item responses. This can be expressed as $p\left( w_{itm}=1|\mathbf{y}_{it} \right)=1$ for state $k$ with the largest $p\left( s_{itm}=1|\mathbf{y}_{it} \right)$. It is important to understand that the assignment of almost any observation includes some amount of uncertainty or “classification error” and can be calculated by conditioning the assigned state membership on the true state membership; that is $p\left( \mathbf{w}_{it} | \mathbf{s}_{itk} \right)$. For details, see S.6.5. As stated before, the amount of classification error is related to the degree of state separation, which is quantified by the entropy-based R-squared measure $R_{entropy}^{2}$. The larger the state separation, the smaller the classification error. In *lmfa*, the state assignments, classification errors, and the $R_{entropy}^{2}$ can be obtained with the step2() function. For details about the arguments and the output, see the Appendix in the main body of the paper.

## Step 3 in *lmfa*

The third step of the 3S procedure consists of estimating the transition model by means of a single indicator CT-LMM (with covariates), which automatically corrects for the classification uncertainty from step 2. More specifically, the single indicator model is:

|  | $p\left( \mathbf{W}_{i}\mathbf{\vert}\mathbf{Z}_{i} \right)=\sum_{\mathbf{s}_{i1}} \cdots\sum_{\mathbf{s}_{iT}} p(\mathbf{s}_{i1}\vert\mathbf{z}_{i1})\prod_{t=2}^{T} p\left( \mathbf{s}_{it} \vert{\mathbf{s}_{it-1}\mathbf{,z}}_{it} \right)\prod_{t=1}^{T} p\left( \mathbf{w}_{it}\mathbf{\vert}\mathbf{s}_{it} \right),$ | (S21) |
| --- | --- | --- |

where the response probabilities $p\left( \mathbf{w}_{it}\mathbf{|}\mathbf{s}_{it} \right)$ are fixed to the classification errors of step 2 and $\mathbf{W}_{i}=\left( \mathbf{w}_{i1},\mathbf{w}_{i2},\ldots,\mathbf{w}_{iT} \right)$ are manifest single indicators (containing error) of the latent (error free) latent states $\mathbf{S}_{i}$ (Di Mari, Oberski, & Vermunt, 2016; Vogelsmeier, Vermunt, Bülow, & De Roover, 2021). Note that $\mathbf{W}_{i}$ and $\mathbf{S}_{i}$ may differ, which is increasingly more likely for larger classification errors. In *lmfa*, the CT-LMM can be estimated with the step3() function. Details about the arguments and the output are given in the Appendix of the paper. In the following, we first show the $\log L$ function that has to be maximized to obtain the ML estimates. Afterward, we describe how the estimation is performed in the *lmfa* package, including the inherent convergence criteria and a multistart procedure.

### Likelihood function

The following $\log L$ function has to be maximized to obtain the ML parameter estimates.

|  | ${\log L}_{STEP3}=\sum_{i=1}^{I} \log\left( p\left( \mathbf{W}_{i}\mathbf{\vert}\mathbf{Z}_{i} \right) \right).$ | (S22) |
| --- | --- | --- |

Note that all parameters have been defined before.

### Estimation

The model is estimated by means of an optimization routine from the *msm* package (Jackson, 2011). The corresponding function msm() can be used to estimate various types of CT-LMMs. In order to obtain the estimates, msm() itself uses the optim() function, which performs “general-purpose optimization” (R Core Team, 2020). In brief, instead of maximizing the $\log L$, optim() minimizes a loss function equal to -2 times the $\log L$. For details about the estimation procedure, we refer to the functions’ documentation, which can be called with the commands ?msm and ?optim. The step3() function in *lmfa* is tailored to the type of model that needs to be estimated in the third step of LMFA (i.e., a single indicator CT-LMM with response probabilities fixed to the classification errors that result from the modal state assignment in step 2). Thus, step3() can be seen as a wrapper that facilitates the usage of msm() by providing proper parameter specifications and constraints and showing the desired parameter estimates, including significance tests for covariate effects using Wald tests.

### Convergence

The optimization of the loss function (S.5.2) stops when the convergence criterion (say, 1e-10, the default) is reached. More specifically, the optimization stops when the loss function (i.e., $-2\log L$) can no further be reduced by a factor equal to the specified tolerance times the sum of the absolute value of the loss function and the tolerance. Thus, when defining the reduction in the loss function as

|  | $\Delta_{-2logL}=$ ${-2log L}_{STEP3}^{v-1}-\left( {-2log L}_{STEP3}^{v} \right)$, | (S23) |
| --- | --- | --- |

the estimation stopes when $\Delta_{-2logL}<1e-10 ({|-2\log L}_{STEP3}^{v}| +1e-10)$.

### Multistart procedure

The results of the CT-LMM are very sensitive to the start values of the log transition intensities. More specifically, as previously stated, the intensities are directly related to the size of the time unit in the dataset. If the unit of the intensities from which the algorithm starts is too far from the actual unit, the model estimation will likely end up in a local maximum. Therefore, the following multistart procedure has been implemented: First, (by default) 25 random diagonal transition probability matrices $\mathbf{P}$ are sampled with staying probabilities on the diagonal that lie between 0.5 and 1. Per row, the off-diagonal probabilities are set equal (considering the constraint that rows must sum to 1). Subsequently, the transition intensity matrices $\mathbf{Q}$ are obtained by taking the matrix logarithm of the transition probability matrices $\mathbf{P}$. Then, the $\mathbf{Q}$ matrix is rescaled by dividing it by the average length of the time intervals. Next, ten initial iterations of the CT-LMM analysis are performed for each start set. The $\log L$ values are obtained and ranked, and the solution with the best $\log L$ value is used for the final analysis. Note that the estimation is not sensitive to the start values of the initial state logits and the covariate effects, which are simply initialized to zero (i.e., covariate effects are absent and probabilities to start in a state are equally likely).

## Statistics

### Number of free parameters measurement models

The number of free parameters for the measurement models obtained in step 1, ${fp}_{STEP1}$, is obtained as follows:

|  | ${fp}_{STEP1}=\overset{state proportions}{\overbrace{K-1}}+\overset{intercepts}{\overbrace{K\times J}}+\overset{unique variances}{\overbrace{K\times J}}+\overset{loadings}{\overbrace{K\times J\times F_{k}}}$. | (S24) |
| --- | --- | --- |

Note that the number of activated constraints for small variances (see S.3.4) would be subtracted from ${fp}_{STEP1}$.

### BIC measurement models

The BIC considers complexity and parsimony by penalizing models with more parameters (${fp}_{STEP1}$) and larger sample size ($N$) as follows:

|  | $\mathrm{BIC}_{STEP1}=-2{\times\log L}_{STEP1}+{fp}_{STEP1}\times log\left( N \right),$ | (S25) |
| --- | --- | --- |

with ${\log L}_{STEP1}$ as in Equation (S5), ${fp}_{STEP1}$ as in Equation (S24), and $N=\sum_{k=1}^{K} N_{k}$ with $N_{k}$ as in Equation (S10).

### CHull scree-test value

For all models (but the least and most complex model) on the upper boundary of the convex hull (CHull), the following scree-test value, $st$, is obtained:^[[3]](#footnote-3)^

|  | ${st}_{n}=\frac{\left( \frac{{\log L}_{STEP1,n}-{\log L}_{STEP1,n-1}}{{fp}_{n}-{fp}_{n-1}} \right)}{\left( \frac{{\log L}_{STEP1,n+1}-{\log L}_{STEP1,n}}{{fp}_{n+1}-{fp}_{n}} \right)},$ | (S26) |
| --- | --- | --- |

where the index $n$ denotes the $n$th hull model. Note that the numerator and the denominator pertain to the slopes of two consecutive parts of the upper boundary of the CHull. Large values of ${st}_{n}$ indicate that model $n$ fits clearly better than model $n-1$, while model $n+1$ only leads to a small increase in model fit (Bulteel et al., 2013).

### Explained variance

The amount of explained variance, $EV$, is calculated by taking the sum of squares of the standardized loadings per state, ${SS}_{k}$, dividing them by the number of items, $J$, weighting them by the state proportions, $p\left( s_{itk}=1 \right)$, and, finally, adding them up across states $K$. Thus,

|  | ${SS}_{k}=\sum_{j=1}^{J} \sum_{f=1}^{F_{k}} \left( \lambda_{kjf} \right)^{2},$ | (S27) |
| --- | --- | --- |
|  | $EV=\sum_{k=1}^{K} {SS}_{k}/J\times p\left( s_{itk}=1 \right).$ | (S28) |

### Classification error

The classification error is obtained by conditioning the assigned state $w_{itm}=1$ on the true, latent state $s_{itk}=1$ for all states $m,k=1,\ldots,K$. That is:

|  | $p\left( w_{itm}=1 \vert s_{itk}=1 \right)=\frac{\frac{1}{I\times T}\sum_{i=1}^{I} \sum_{t=1}^{T} p\left( w_{itm}=1\vert\mathbf{y}_{it} \right)p\left( s_{itk}=1\vert\mathbf{y}_{it} \right)}{p\left( s_{itk}=1 \right)}.$ | (S29) |
| --- | --- | --- |

For a derivation, see Vogelsmeier et al. (2021). Note that $p\left( s_{itk}=1 \right)$ are the state proportions (Equation (S11) and $p\left( w_{itm}=1|\mathbf{y}_{it} \right)$ are the modal state assignments based on the posterior state-membership probabilities $p\left( s_{itk}=1|\mathbf{y}_{it} \right)$ (Equation (S9).

### R-squared entropy

The state separation in terms of the R-square measure $R_{entropy}^{2}$ can be calculated as follows:

|  | $R_{entropy}^{2}=\frac{Entropy\left( \mathbf{S} \right)-Entropy(\mathbf{S}\vert\mathbf{Y})}{Entropy\left( \mathbf{S} \right)}=1-\frac{Entropy(\mathbf{S}\vert\mathbf{Y})}{Entropy\left( \mathbf{S} \right)}$ | (S30) |
| --- | --- | --- |

with

|  | $Entropy\left( \mathbf{S} \right)=\sum_{i=1}^{I} \sum_{t=1}^{T} \sum_{k=1}^{K} -p\left( s_{itk}=1 \right)\log p\left( s_{itk}=1 \right)$ | (S31) |
| --- | --- | --- |

and

|  | $Entropy\left( \mathbf{S} \vert\mathbf{Y} \right)=\sum_{i=1}^{I} \sum_{t=1}^{T} \sum_{k=1}^{K} -p\left( s_{itk}=1\vert\mathbf{y}_{it} \right)\log p\left( s_{itk}=1\vert\mathbf{y}_{it} \right).$ | (S32) |
| --- | --- | --- |

Thus, Equation (S30) shows that the $R_{entropy}^{2}$ measure determines the relative improvement in predicting the state memberships given the observations (numerator) versus predicting the state memberships without the observations (denominator).

### Wald-test statistic

The Wald statistic $W^{\mathbf{2}}$ is computed as follows:

|  | $W^{\mathbf{2}}\boldsymbol{=}\theta_{u}^{\boldsymbol{'}}{\hat{\Sigma}\left( \theta_{u} \right)}^{-1}\theta_{u}$ | (S33) |
| --- | --- | --- |

with $\theta_{u}$ and ${\hat{\Sigma}\left( \theta_{u} \right)}^{-1}$ indicating the slopes and the estimated variance covariance matrix for covariate $u$, respectively. Note that the degrees of freedom (df) are equal to the number of constrained parameters (e.g., df = 6 when testing the significance of a covariate with six slopes).

### Number of free parameters transition models

The number of free parameters, ${fp}_{STEP3}$, is obtained as follows:

|  | ${fp}_{STEP3}=\overset{initial state probabilities}{\overbrace{\left( K-1 \right)}\times\left( 1+U_{ini} \right)}+\overset{transition probabilities}{\overbrace{\left( K^{2}-K \right)}\times\left( 1+U_{tra} \right)},$ | (S34) |
| --- | --- | --- |

with $U_{ini}$ referring to the number of covariates for the initial state probabilities and $U_{tra}$ referring to the number of covariates for the transition probabilities.

### BIC transition models

The BIC considers complexity and parsimony by penalizing models with more parameters (${fp}_{STEP3}$) and larger sample size ($N$) as follows:

|  | $\mathrm{BIC}_{STEP3}=-2{\times\log L}_{STEP3}+{fp}_{STEP3}\times log\left( N \right),$ | (S35) |
| --- | --- | --- |

with ${\log L}_{STEP3}$ as in Equation (S22), ${fp}_{STEP3}$ as in Equation (S34), and $N=\sum_{k=1}^{K} N_{k}$ with $N_{k}$ as in Equation (S10).

### Factor scores

The state-specific factor score estimates, ${\hat{\mathbf{F}}}_{ik}$, are obtained by means of the regression method (Thomson, 1934; Thurstone, 1935):

|  | ${\hat{\mathbf{F}}}_{ik}=\mathbf{Y}_{i}{\boldsymbol{\Sigma}_{\boldsymbol{k}}^{\boldsymbol{-1}}\boldsymbol{\Lambda}}_{k}\boldsymbol{\Phi}_{k}$, | (S36) |
| --- | --- | --- |

with $\boldsymbol{\Sigma}_{k}=\boldsymbol{\Lambda}_{k}\boldsymbol{\Phi}_{k}\boldsymbol{\Lambda}_{k}^{'}+\boldsymbol{\Psi}_{k}$.

# References

Bartolucci, F., Farcomeni, A., & Pennoni, F. (2014). Comments on: Latent Markov models: a review of a general framework for the analysis of longitudinal data with covariates. *Test, 23*, 473–477. doi:10.1007/s11749-014-0387-1

Bulteel, K., Wilderjans, T. F., Tuerlinckx, F., & Ceulemans, E. (2013). CHull as an alternative to AIC and BIC in the context of mixtures of factor analyzers. *Behavior Research Methods, 45*, 782–791. doi:10.3758/s13428-012-0293-y

Cox, D. R., & Miller, H. D. (1965). *The Theory of Stochastic Process*. London: Chapman & Hall.

Di Mari, R., Oberski, D. L., & Vermunt, J. K. (2016). Bias-adjusted three-step latent Markov modeling with covariates. *Structural Equation Modeling: A Multidisciplinary Journal, 23*, 649–660. doi:10.1080/10705511.2016.1191015

Jackson, C. H. (2011). Multi-state models for panel data: The msm package for R. *Journal of Statistical Software, 38*, 1–29.

McLachlan, G. J., & Peel, D. (2000). Mixtures of factor analyzers. In P. Langley (Ed.), *Proceedings of the Seventeenth International Conference on Machine Learning.* (pp. 599–606). San Francisco: Morgan Kaufmann.

McNicholas, P. D. (2016). *Mixture Model-Based Classification*. Boca Raton: Chapman and Hall/CRC Press.

R Core Team. (2020). A language and environment for statistical computing. R Foundation for Statistical Computing. Vienna, Austria.

Scrucca, L., Fop, M., Murphy, T. B., & Raftery, A. E. (2016). mclust 5: clustering, classification and density estimation using Gaussian finite mixture models. *The R journal, 8*, 289–317.

Thomson, G. H. (1934). The meaning of i in the estimate of g. *British Journal of Psychology, 25*, 92–99. doi:10.1111/j.2044-8295.1934.tb00728.x

Thurstone, L. L. (1935). *The vectors of mind*. Chicago, IL: University of Chicago Press.

Tipping, M. E., & Bishop, C. M. (1999). Probabilistic principal component analysis. *Journal of the Royal Statistical Society, 61*, 611–622. doi:10.1111/1467-9868.00196

Vermunt, J. K., Langeheine, R., & Böckenholt, U. (1999). Discrete-time discrete-state latent markov models with time constant and time-varying covariates. *Journal of Educational and Behavioral Statistics, 24*, 179–207. doi:10.2307/1165200

Vervloet, M., Wilderjans, T. F., Durieux, J., & Ceulemans, E. (2017). *multichull: A Generic Convex-Hull-Based Model Selection Method*.

Vogelsmeier, L. V. D. E., Vermunt, J. K., Bülow, A., & De Roover, K. (2021). Evaluating covariate effects on ESM measurement model changes with latent Markov factor analysis: A three-step approach. *Multivariate Behavioral Research*. doi:10.1080/00273171.2021.1967715

1. Note that the number of time-points $T$ may differ across subjects but we mostly omit the index $i$ for the sake of simplicity. [↑](#footnote-ref-1)
2. Note that this value cannot be changed by the user. [↑](#footnote-ref-2)
3. For the procedure to obtain the models on the upper boundary, see Bulteel, Wilderjans, Tuerlinckx, and Ceulemans (2013) and Vervloet, Wilderjans, Durieux, and Ceulemans (2017). [↑](#footnote-ref-3)
